# Gut microbiota of homing pigeons shows summer–winter variation under constant diet indicating a substantial effect of temperature

**DOI:** 10.1186/s42523-022-00216-6

**Published:** 2022-12-13

**Authors:** Maurine W. Dietz, Kevin D. Matson, Maaike A. Versteegh, Marco van der Velde, Henk K. Parmentier, Joop. A. J. Arts, Joana F. Salles, B. Irene Tieleman

**Affiliations:** 1 Groningen Institute for Evolutionary Life Sciences, University of Groningen, Nijenborgh 7, 9747AG Groningen, The Netherlands; 2 Wildlife Ecology and Conservation, Environmental Science Group, Wageningen University & Research, Droevendaalsesteeg 3a, 6708PB Wageningen, The Netherlands; 3 Adaptation Physiology Group, Department of Animal Sciences, Wageningen University & Research, De Elst 1, 6708 WD Wageningen, The Netherlands

**Keywords:** Avian microbiota, Basal metabolic rate, Day length, Host-microbiota interactions, Immune competence, Season

## Abstract

**Background:**

Gut microbiotas play a pivotal role in host physiology and behaviour, and may affect host life-history traits such as seasonal variation in host phenotypic state. Generally, seasonal gut microbiota variation is attributed to seasonal diet variation. However, seasonal temperature and day length variation may also drive gut microbiota variation. We investigated summer–winter differences in the gut bacterial community (GBC) in 14 homing pigeons living outdoors under a constant diet by collecting cloacal swabs in both seasons during two years. Because temperature effects may be mediated by host metabolism, we determined basal metabolic rate (BMR) and body mass. Immune competence is influenced by day length and has a close relationship with the GBC, and it may thus be a link between day length and gut microbiota. Therefore, we measured seven innate immune indices. We expected the GBC to show summer–winter differences and to correlate with metabolism and immune indices.

**Results:**

BMR, body mass, and two immune indices varied seasonally, other host factors did not. The GBC showed differences between seasons and sexes, and correlated with metabolism and immune indices. The most abundant genus (*Lachnoclostridium *12, 12%) and associated higher taxa, were more abundant in winter, though not significantly at the phylum level, *Firmicutes*. *Bacteroidetes* were more abundant in summer. The *Firmicutes*:*Bacteroidetes* ratio tended to be higher in winter. The KEGG ortholog functions for fatty acid biosynthesis and linoleic acid metabolism (PICRUSt2) had increased abundances in winter.

**Conclusions:**

The GBC of homing pigeons varied seasonally, even under a constant diet. The correlations between immune indices and the GBC did not involve consistently specific immune indices and included only one of the two immune indices that showed seasonal differences, suggesting that immune competence may be an unlikely link between day length and the GBC. The correlations between the GBC and metabolism indices, the higher *Firmicutes*:*Bacteroidetes* ratio in winter, and the resemblance of the summer–winter differences in the GBC with the general temperature effects on the GBC in the literature, suggest that temperature partly drove the summer–winter differences in the GBC in homing pigeons.

**Supplementary Information:**

The online version contains supplementary material available at 10.1186/s42523-022-00216-6.

## Background

Animals can anticipate and respond to changes in specific environmental conditions. A significant driver of environmental changes is seasonal variation in climate, which can be expected from highly predictable cues like day length. Seasonal abiotic factors are often associated with changes in different facets of organismal biology, such as reproductive and physiological state, food abundance, and behaviour. Animals may rely on predictable cues like day length, their endogenous annual pacemakers (internal mechanisms that govern annual biological rhythms), or both to respond to predictable seasonal environmental variation [1, 2]. As a result, the annual life cycles of animals, characterized by changes in behavioural, physiological, and morphological phenotypes [2–6], are predictably timed and maintained in captivity [5, 7].

The microbiota (i.e., bacteria, archaea, lower and higher eukaryotes, and viruses [8]) living in and on individuals can also influence the physiology and behaviour of animals [9, 10]. Because we, just as the vast majority of studies on microbiota, focus on the bacterial part of the microbiota, we will further use “gut bacterial community” instead of gut microbiota to indicate that we refer to the bacterial part of the gut microbiota. The gut bacterial community is one of the largest animal microbial communities in number of species as well as biomass, and its symbiotic relationships with the hosts are often complex and bidirectional [10]. For example, the diet choice of hosts can strongly affect the composition and function of the hosts’ gut bacterial community. At the same time, the gut bacterial community may influence diet selection [9–11] as shown by transplantation experiments in germ-free mice [12]. After transplantation, the bacterial genes related to tryptophan metabolism correlated with diet choice in these mice, supporting the hypothesis that the gut bacterial community may influence diet choice and food intake via the metabolization of tryptophan [13]. Because the gut bacterial community influences host physiology and because there may also be a link between gut bacterial community and behaviour [14–16], the gut bacterial community contributes to the phenotypic flexibility of their hosts [10, 17] and thus may assist hosts in responding to seasonal changes. Hence, the gut bacterial community is expected vary seasonally, as it does in some wild animals [18–21]. This seasonal variation in the gut bacterial community seems to be primarily driven by seasonal variation in diet [22]. In North American red squirrels (*Tamiasciurus husonicus*), for example, seasonal rhythms in the relative abundances of *Oscillospira* and *Corpococcus* genera were associated with seasonal variation in food availability [18]. While in the greater sage-grouse (*Centrocercus urophasianus*), seasonal variation in food quality likely explains the seasonal variation in gut bacterial community composition and richness, possibly in combination with the seasonal variation in food- and water-associated microbiota [21].

In addition to diet, two seasonally varying abiotic factors may contribute to the seasonal variation in gut bacterial community: temperature and day length [23–26]. The gut bacterial community composition and function can change rapidly with changing temperature. For example, the gut bacterial community of captive mice (*Mus musculus*) and Eastern red-backed salamanders (*Plethodon cinereus*) changed in microbial diversity, community composition, and relative abundances of different taxa after the animals were housed at low temperatures for 7–11 days [27–29]. In many host species, the effects of temperature change on gut bacterial community are reflected in a variation in the relative abundances of different taxa. In vertebrates, *Firmicutes* are generally more abundant at lower ambient temperatures, while *Bacteroidetes* are more abundant at higher temperatures [24]. How ambient temperature directly affects the gut bacterial community is unclear, but mechanisms related to host metabolism are likely to play a role. For instance, temperature influences host metabolism via changing thermoregulation costs (independently of its gut bacterial community), and the changing metabolism may shape the gut bacterial community [23]. Transfers of the gut bacterial community (via co-housing and caecal or faecal transplantations) from hosts that were cold-acclimatized to those that were not induced changes in the metabolism and nutrient assimilation of the recipients. These physiological changes, including the promotion of browning of white fat depots and the elevation of metabolic rate [24, 27, 28, 30, 31], mimicked changes due to cold-acclimatization. This suggests that seasonal temperature differences may result in a seasonal variation in the gut bacterial community that helps drive the seasonal acclimatization of hosts.

Seasonal variation in the gut bacterial community may also relate to variation in day length or the light–dark cycle. Day length variation affects the circadian clocks of animal hosts, leading to, for example, seasonal acclimatization [25, 26, 32]. The relationships between the host’s circadian systems and the gut bacterial communities are very complex and bidirectional. The importance of the host circadian clock in maintaining circadian rhythms in the gut bacterial community can be seen in Per1/2 mice (*Mus musculus*). These mice lack the essential clock genes Per1 and Per2 that drive the daily oscillations of the master pacemaker in the brain, and these mice have lost the diurnal oscillations in the total number (i.e., total number of bacterial cells) of mucosal-resident bacteria [33]. The circadian oscillations in the gut bacterial community can be restored in these mice through time-restricted feeding [33, 34]. Daily rhythms in hosts can also govern the effects of the gut bacterial community on other physiological systems of the hosts. For example, the host's circadian system mediates the vital communication between the gut bacterial community and the host’s immune system [26]. Likewise, daily rhythms in the gut bacterial community can affect hosts: e.g., bacterial community-produced short-chain fatty acids and bile acids can induce circadian entrainment in certain tissues and modulate hepatic circadian gene expression in mice [32]. Most research to date has focused narrowly on the direct effects of day length on the daily rhythm in the gut bacterial community characteristics. It remains unclear whether seasonal patterns in day length result in corresponding seasonal patterns in the gut bacterial community and whether this interaction aids hosts in adjusting to seasonal environmental variation.

**Fig. 1:**
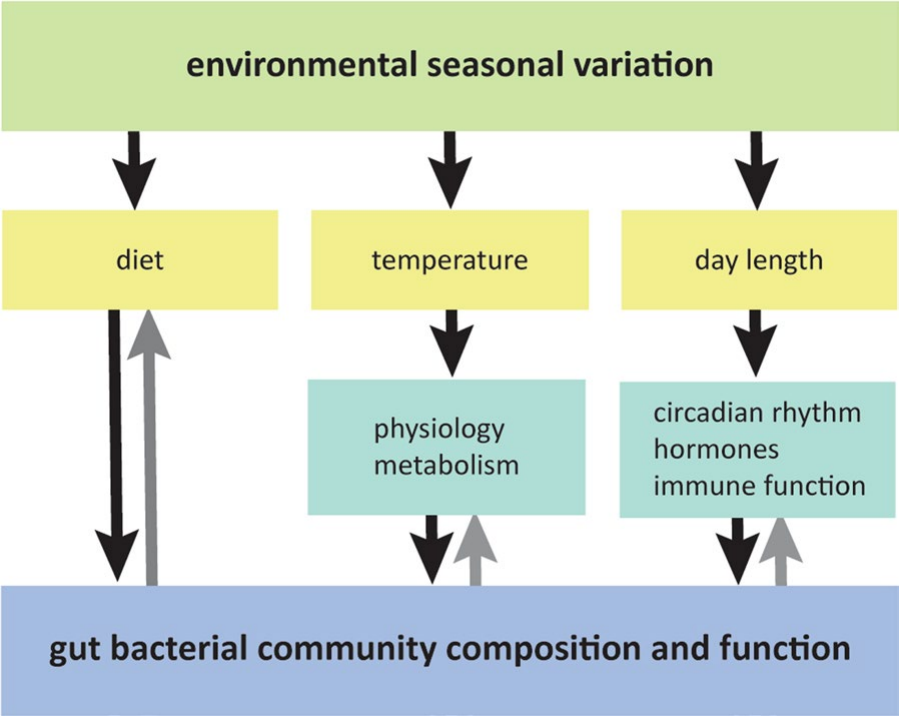
Schematic overview of direct and indirect pathways via which season may impact the gut bacterial community. Yellow boxes indicate the three important aspects of seasonal environmental variation (diet, temperature, day length) that may impact the gut bacterial community. Green boxes indicate the host components of the indirect pathways. E.g., seasonal day length variation impacts the circadian system of the host, which will impact the gut bacterial community composition and function via seasonal variation in feeding patterns and circadian rhythms in hormone expression. On its turn, the gut bacterial community (blue box) impacts the host circadian system short chain fatty acids and bile acids production. Black arrows indicate the route of seasonal pathways modulating the gut bacterial community, grey arrows indicate modulation of the host by the gut bacterial community

Via host-mediated effects, seasonal variation in temperature and day length may contribute to the seasonal variation in the gut bacterial community, above and beyond any effect of seasonal changes in diet (Fig. 1). To explore this possibility, we investigated the summer–winter differences in the gut bacterial community in relation to host physiology in homing pigeons (*Columba livia*) that were fed a constant diet. During summer and winter of two consecutive years, we collected cloacal swabs and other host-related data from 14 individuals (six females and eight males) housed in outdoor aviaries. Because diet was constant, summer–winter differences in the gut bacterial community may be partly attributed to temperature and day length variation. As host metabolism may mediate the effects of temperature on the gut bacterial community, we investigated whether summer–winter variation in host metabolism was correlated with summer–winter variation in the gut bacterial community. To do so, we quantified basal metabolic rate (BMR) and body mass during both years, and daily food intake and digestive efficiency during summer and winter of the first year. In addition, we compared our results with the general effects of temperature variation on the gut bacterial community found in the literature. The link between day length and the gut bacterial community may be immune competence, because day length may affect the immune system and via the circadian clock also the communication between the immune system and the gut bacterial community. Therefore, we assessed seven indices of the innate immune system during both years. We expected (1) that the gut bacterial community would show summer–winter differences despite a constant ad libitum food source; (2) that *Firmicutes* would be relatively more abundant in the winter due to lower temperatures, and *Bacteroidetes* would be relatively more abundant in summer cf. [24]; (3) that metabolic differences would parallel summer–winter differences in the gut bacterial community since temperature affects host metabolism, and host metabolism influences the gut bacterial community [23]; and (4) that immunological differences would parallel summer–winter differences in the gut bacterial community since day length may directly affect the immune system and mediate, via the host’s circadian clock, the communication between gut bacterial community and the immune system [26].

## Methods

### Animals

We used 14 homing pigeons (six females and eight males) hatched in captivity in late 2005, housed in same sex groups of 2–4 individuals in outdoor aviaries (4.01 m × 1.67 m × 2.2 m, l × w × h) at the Groningen Institute for Evolutionary Life Sciences (GELIFES) of the University of Groningen (N53°14.579’ E6°32.271’). Food (seed mixture 4 seasons for homing pigeons KASPER™ 6705, and pigeon pellets KASPER™ P40, Kasper Faunafood, Woerden, Netherlands; see Additional file 1: Table S1 for composition), grit and water were available ad libitum. All birds were exposed to outside air temperature and natural day length (see Table 1 for summer–winter differences in the experimental years). The birds were colour banded for individual identification.

**Table 1:** Seasonal variation in day length, temperature, body mass, BMR, immune indices, diet and digestive efficiency

Factors *	Sex	Summer 2013	Winter 2014	Summer 2014	Winter 2015
Temperature ^a^		18.3 ± 2.4	4.0 ± 3.8	19.7 ± 2.8	3.5 ± 3.1
Day length ^b^		16.3 ± 0.4	8.2 ± 0.4	16.3 ± 0.4	8.2 ± 0.4
Body mass	F	526.0 ± 38.0	516.3 ± 34.4	511.1 ± 39.2	565.9 ± 26.4
	M	498.4 ± 60.9	636.3 ± 44.2	505.1 ± 50.0	621.1 ± 44.3
BMR	F	368.8 ± 34.5	408.5 ± 28.0	368.7 ± 34.5	362.1 ± 14.4
	M	328.0 ± 59.3	450.7 ± 52.3	343.1 ± 51.7	403.1 ± 32.4
HP(res)	F	0.001 ± 0.055	0.024 ± 0.070	− 0.024 ± 0.023	0.042 ± 0.085
	M	0.015 ± 0.040	0.001 ± 0.051	− 0.009 ± 0.18	− 0.040 ± 0.023
HL(res)	F	− 0.05 ± 0.99	− 0.22 ± 0.45	0.29 ± 0.95	0.40 ± 0.67
	M	0.42 ± 0.97	− 0.24 ± 00.67	− 0.48 ± 0.81	0.24 ± 0.81
HG	F	7.69 ± 0.06	7.48 ± 0.10	8.70 ± 0.08	8.79 ± 0.05
	M	8.61 ± 0.06	6.91 ± 0.06	7.00 ± 0.06	8.29 ± 0.06
KLH	F	4.9 ± 0.9	4.7 ± 1.1	5.4 ± 1.6	5.3 ± 1.0
	M	4.5 ± 0.8	4.2 ± 0.7	4.6 ± 1.0	4.3 ± 0.8
HuSA	F	4.1 ± 2.7	3.9 ± 2.0	5.3 ± 1.7	4.8 ± 2.2
	M	3.1 ± 1.3	3.5 ± 1.6	4.8 ± 2.5	4.4 ± 2.3
BSA	F	4.3 ± 1.5	4.1 ± 1.7	5.6 ± 3.6	4.5 ± 3.2
	M	2.7 ± 1.8	2.5 ± 1.7	3.2 ± 2.2	3.6 ± 2.5
PC-BSA	F	2.5 ± 2.2	3.5 ± 1.3	4.0 ± 2.1	3.5 ± 1.4
	M	3.7 ± 1.3	2.7 ± 1.2	3.2 ± 1.4	2.8 ± 1.6
Total consumption	F	20.6 ± 13.9	21.5 ± 12.4		
	M	20.1 ± 4.4	18.7 ± 7.2		
Pellet consumption	F	79.4 ± 21.8	82.3 ± 26.3		
	M	44.8 ± 36.3	47.1 ± 31.2		
Digestive efficiency	F	0.72 ± 0.07	0.64 ± 0.18		
	M	0.77 ± 0.08	0.73 ± 0.05		

*Factors and units: temperature is the mean daily temperature averaged over July or January (°C); day length averaged over July or January (hr); body mass was determined when the cloacal swab was taken (g), BMR is basal metabolic rate (ml O_2_∙h^−1^); the innate immune indices are: HP(res), residual haptoglobin concentration (mg ml^−1^), HL(res), residual haemolysis, HG, hemagglutination, KLH, keyhole limpet hemocyanin, HuSA, human serum albumin, BSA, bovine serum albumin, and PCBSA, phosphorylcholine conjugated to BSA (unit latter six indices: antibody titres against the immune indices); total consumption: the total amount of food eaten (g); pellet consumption: the percentage pellets in the diet; and the digestive efficiency, i.e., the assimilation quotient. Presented are mean and SD between brackets for summers and winters in 2013–2015. Sample sizes were 6 females (F) and 8 males (M), except for summer 2013 when we had data of 7 males. For significant differences, see text and Table S2 in the Additional File. ^a^Temperature data was determined at the weather station 280 of the Royal Netherlands Meteorological Institute (KNMI), located at Eelde, ca. 13 km south of the aviaries (N 53° 7.674′, E 6° 35.152′; data is available at https://www.knmi.nl/nederland-nu/ klimatologie/daggegevens). ^b^Day length data was obtained from https://www.sunrise-and-sunset.com/nl/sun/nederland/groningen

### Cloacal swab collection

**Fig. 2:**
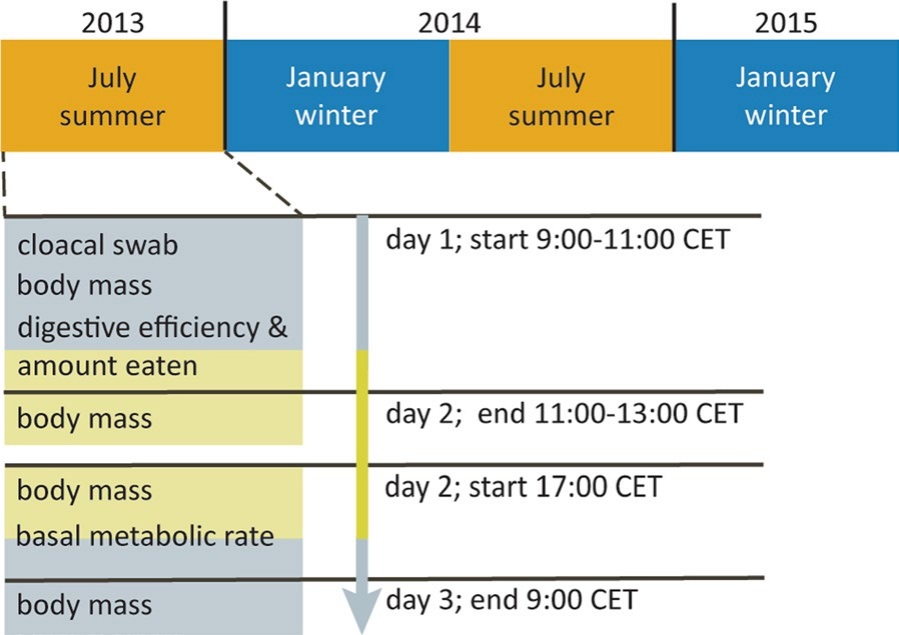
Schematic overview of the experimental set-up. Sampling months are given in the top of the figure. The experimental set-up per sampling month is given in detail for the first sampling point in the lower part of the figure. Indicated are the order, type of sample and data collected, experimental day, and start and end time

We collected cloacal swabs in July (summer) and January (winter) between 9:00 and 11:00 CET for two consecutive years, starting in July 2013, see Fig. 2 for the experimental set-up. We inserted a sterile viscose swab into the cloaca without contacting feathers or skin and gently rotated it for 10 s in the intestinal lumen. Swab tips, which were cut from the shaft using scissors sterilized with 76% ethanol then flamed, were stored in a sterilized 1.5 ml vials. After adding a drop of sterile PBS, swabs were stored at − 20 °C until analysis. We randomized the sampling order of the individual pigeons per season and sampled two pigeons per day (rate limited by further handling protocols describes below). Per sampling month, one cloacal swab was collected from each individual (Fig. 2). Body mass was recorded after swab collection (± 0.1 g).

### Daily food intake and digestive efficiency

We determined daily food intake and digestive efficiency after cloacal sampling, and placed for this the pigeons individually in clean outdoor aviaries located in the same aviary cluster as their home aviary. Each bird was offered ~ 50 g of pellets, ~ 60 g of wheat, ~ 30 g of corn, and ~ 30 g of green peas (the latter three are the main seeds provided and eaten from the seed mixture offered), and ad libitum water. The next day between 11:00 and 13:00 CET, we removed the birds from the aviaries, recorded body mass again, and transferred them indoors for the basal metabolic rate measurement. All faeces and food leftovers were collected and weighed. Faeces were stored at − 20 °C until analysis.

We determined the energy and water contents once for each food item (pellets, wheat, corn, and green peas) and used these data to calculate dry food mass eaten and energy intake for each food item per trial. Faecal energy content was determined per individual trial. Before determining energy contents, we dried the food items and faeces to constant mass at 60 °C, i.e., until the change between weightings was < 0.1% of the initial fresh mass (all masses ± 0.0001 g). This took ~ 13 d for food items and ~ 6 d for faeces. We ground dry food and faeces to powder (Retsch grinder ZM 100), pressed them into pills (~ 1 g), and dried them to constant mass at 60 °C to determine pill dry mass (± 0.0001 g). We burned the pills in an adiabatic bomb calorimeter (IKA C 5000) to determine their energy content (kJ∙g^−1^). We analysed all samples at least in duplicate, which in general differed by < 2% of the lower energy content pill. Two samples were measured in triplicate. The mean energy content of the replicates was used in further analyses. For each trial, the digestive efficiency or assimilation quotient was calculated as:

$$\frac{{\left( {sum\left( {E\;food\;item_{i} *dry\;mass\;consumed\;food\;item_{i} } \right)} \right) - \;\left( {E\;faeces*dry\;mass\;faeces\;produced} \right)}}{{sum\left( {E\;food\;item_{i} *dry\;mass\;consumed\;food\;item_{i} } \right)}}$$

where *E* is the energy content of the dry *food item*_*i*_ or faeces, and *food item*_*i*_ is pellets, wheat, corn, or green peas.

During the first year, pigeons ate typically > 20 g food (summer 2013 23.16 ± 10.28 g, winter 2014 20.09 ± 12.15 g), while in the second year, almost all pigeons ate much less (summer 2014 7.88 ± 6.23 g, winter 2015 5.51 ± 7.93 g). Hence, we disregarded the second year's data. The lower food consumption in the second year was unexpected, and we cannot explain this observation as generally repeating procedures with animals is expected to result in less stress. In the second year, we did determine the food intake of a few pigeons left isolated in their home cage. Food amounts eaten by these birds were similar to the 2013–2014 values, suggesting that not isolation itself but rather the aviary transfer in combination with isolation led to the lower food consumption in the second year.

### Basal metabolic rate

Prior to measuring BMR, the pigeons were placed individually in a darkened box (30 cm × 25 cm × 28 cm) indoors to acclimatize and fast for ~ 4 h. At ~ 17:00 CET, the birds were placed individually into 13.5 l metabolic chambers and placed inside a climatic chamber set at 25 ± 0.5 °C (thermoneutral for domestic pigeons [35, 36]). Oxygen consumption was measured throughout the night using standard flow-through respirometry methods and recorded during 17-min windows alternately for each individual (for details, see [37]). The following day at ~ 9:00 CET, the bird was removed from the metabolic chamber and returned to its aviary. Body mass was recorded immediately before and after the measurement. BMR (ml O_2_∙h^−1^) was based on the lowest average oxygen consumption during any of the 17-min windows recorded throughout the night.

### Immune indices

We assessed innate immune competence from blood samples collected ca. one month prior to each microbiota sampling moment. We measured seven innate immune indices using three assay types. First, we used a commercially available colorimetric assay (TP801; Tri-Delta Diagnostics, NJ, USA) to quantify haptoglobin concentration (or its haem-binding functional equivalents, mg∙ml^−1^). We followed the manufacturer’s instructions with the additions and changes described in [38]. Because this functional assay is sensitive to contamination by haem in haemolysed samples, we measured sample redness (absorbance at 450 nm), a proxy for haemolysis, prior to the addition of the second reagent and the initiation of the colour change reaction [38]. In the current dataset, the relationship between sample redness and haptoglobin was significant (*P* = 0.02), so we used the residual variation in haptoglobin in further analyses. Haptoglobin did not vary with sample age.

Second, we used a haemolysis-haemagglutination assay to measure titres of complement-mediated lysis, and natural antibody- (NAb-) mediated agglutination of rabbit erythrocytes [39]. Agglutination was recorded from plate images made 20 min post-incubation; lysis was recorded from plate images made 24 h after incubation, as described in [40]. In the current dataset, the relationship between sample age and lysis (but not sample age and agglutination) was significant (agglutination, *P* = 0.82; lysis, *P* < 0.01); hence we used the residual variation in lysis in further analyses.

Third, we used indirect three step ELISA to measure titres of NAbs against four antigens separately, none of which individuals had been previously vaccinated against: keyhole limpet hemocyanin (KLH), human serum albumin (HuSA), bovine serum albumin (BSA), and phosphorylcholine conjugated to BSA (PC-BSA) [41]. In brief, wells were incubated with 100uL of coating buffer (pH 9.6) containing one of the four antigens for one hour at 37 °C. Wells were then washed (water + 0.05% Tween 20), blocked (phosphate buffer saline (PBS) + 1% horse serum + 0.05% Tween 20) for 30 min, and washed again. Plasma samples were serially four step diluted (KLH: 1:40, 1:160, 1:640, 1:2560; other antigens two step dilutions: 1:20, 1:40, 1:80, 1:160) in wells containing 100uL dilution buffer (PBS + 0.5% horse serum + 0.05% Tween 20). Duplicate standard positive plasma samples (a pool of pigeons) were two step diluted with dilution buffer. Two antibodies were added and incubated (1 h at 37 °C) sequentially: first, 100 μL of a 1:5000 dilution of rabbit-anti-pigeon antibodies (IgG(H + L); Nordic; batch no. 6162); second, 100 μL of a 1:2000 swine-anti-rabbit antibodies conjugated to horseradish peroxidase. After each incubation, wells were washed. The colour change reaction was initiated with the addition of substrate (containing reverse osmosis purified water, 10% tetramethylbenzidine buffer [15.0 g/L sodium acetate, and 1.43 g/L urea hydrogen peroxide; pH 5.5], and 1% tetramethylbenziding [8 g/L TMB in DMSO]) at room temperature and stopped (with 50 μl/well of 1.25 M H_2_SO_4_) after 15 min; absorbance was read at 450 nm with a Multiskan Go (Thermo scientific). All titres of the NAbs were not correlated with sample age (all *P* > 0.1). Antibody titres were calculated as described by [42] (taken from [43]). For details on the antibody titres calculation see the Supporting Information.

### DNA isolation and 16S rRNA gene amplicon sequencing

We randomized the cloacal swabs prior to DNA extraction. DNA was isolated from the samples using the FastDNA™ kit for Soil (MP Biomedicals, Santa Ana, CA, USA) according to the manufacturer’s instructions. Two exceptions were cell lysis, which was achieved by beat-beating three times one minute instead of three minutes continuously, to prevent the samples from heating up, and the DNA elution which was done using in 100 µl PCR-grade water. We quantified sample DNA concentrations using the Quant-it PicoGreen dsDNA kit (Molecular Probes, Invitrogen, Eugene, OR, USA) and normalized the DNA concentrations in the subsequent PCR to 1 ng template DNA per 25 μl reaction. The samples were randomized again before amplifying the V4/V5 region of the 16S rRNA gene in a triplicate using the primers 515F and 926R [44, 45] with Illumina adaptors at the 5’-end. We used the following thermal cycling protocol: 5 min at 95 °C, 35 cycles with 40 s at 95 °C, 45 s at 56 °C, 40 s at 72 °C, followed by 10 min at 72 °C. We pooled the triplicates after the PCR. We excluded one cloacal sample with poor PCR results (a male, summer 2013), and sent after purification (QIAquick gel extraction Kit, QIAGEN GmbH, Hilden, Germany) the 55 pigeon samples, a negative control swab and 4 negative PCR controls, to GenoToul (INRA, Toulouse, France) for library preparations and Illumina sequencing using 2 × 250 bp v2 chemistry. At GenoToul, the sequence reads were demultiplexed and quality filtered using the default settings in QIIME.

### Sequence data processing

We processed the raw sequence data using the standard QIIME2 protocol (v2018.2 [46]). Using the DADA2 (v2018.2) pipeline, we trimmed the primers, truncated the forward and reverse reads to 250 bp and 190 bp, respectively, merged the forward and reverse reads based on quality plots (at least 25 bp overlap), and removed chimera. The taxonomy table was built using the Silva v132 reference database [47, 48]. Next, we filtered *Archaea*, chloroplasts, mitochondria, and vertebrates from the data. The end products, an Amplicon Sequence Variant (ASV) table and the phylogenetic tree were further processed in R (v4.0.2 [49]) using *Phyloseq* (v1.32.0 [50]) and *vegan* (v2.5-6 [51]). At this stage, the data included 1056 taxa, and the total number of sequence reads was 1,606,609, with counts ranging between 1779 and 95,837 reads for cloacal swab samples and between 78 and 1449 reads for the negative controls.

### Statistical analysis

#### Host parameters

We used linear mixed models (LMM, *nlme* v3.1-148 [52]) to identify correlations between the host parameters (metabolic and immune indices) and season (summer vs. winter), sex, their interaction term (fixed factors), and individual bird colour bands (BirdID) nested within aviary (random factors). Sex was included as physiological and potential diet differences between the sexes may affect the host parameters. The interaction term season*sex was included in the model because seasonal variation may trigger different responses in the two sexes, such as sexual differences in hormonal responses to seasonal variation. We used a stepwise backward exclusion of nonsignificant fixed factors. At each step of the analysis, the normality of the model and homoskedasticity of the residuals were checked. For the final model, we tested if aviary as a random factor contributed significantly (ANOVA), which was never the case. Hence the final model contained only BirdID as random factor.

#### Contamination

We used *Decontam* (v1.8.0 [53]) to identify general contaminants via an objective method, using the recommended settings. The *Decontam* frequency method identified only eight out of the 1056 ASVs as contaminants. However, two of the eight were not present in the negative controls. The *Decontam* prevalence method identified only six contaminants. However, three of the six contaminants occurred in only one cloacal swab sample. For unknown reasons our data seem to challenge the Decontam frequency and prevalence methods. A possible explanation may be the high (though not an exceptionally high) percentage of ASVs with a prevalence of 1 (see below). Given these results, and considering the very low read counts of the negative controls (78–1449 reads), we concluded that no contaminants with a considerable impact on the data could be detected. We therefore removed the reads from negative control samples from the data set. Hereafter the data included 1032 taxa divided over 55 samples, with in total 1,602,954 reads.

#### Sequence data checks and transformation

We checked the data for rare ASVs based on read counts and prevalence. Since we used DADA2 to trim primers and merge and truncate primers, the data initially contained no singletons. After removing the negative control samples, there were 5 singleton ASVs (0.5%) and 185 doubleton ASVs (17.9%), indicating that only few ASVs had low read counts. Prevalence analysis showed that despite having four samples per individual, 81.3% of the ASVs occurred in only 1 sample, indicating a high variability between the samples within and among individuals. This percentage is comparable to other data sets of ours, including a dataset from captive juvenile rock pigeons that includes eight samples per individual (77.8%) and a dataset from adult free-living feral pigeons (61.3%) (Dietz pers. comm.). These percentages are also comparable to those from a variety of other species [54].

There are multiple ways to transform sequence data prior to analyses, each with pros and cons. We tested four methods: (1) rarefying [56, 57], (2) proportional transformation, i.e., total sum scaling (TSS) [55–58], (3) centered log ratio transformation (clr) [59, 60], and (4) a DESeq2 transformation [61]. The results for the alpha- and beta-diversity analyses were comparable for these methods. Therefore, we proceeded with the most commonly used method, rarefying. Because richness rarefactions curves levelled off around 3,000 reads (see Additional file 1: Fig. S1), we rarefied the data to 3270 reads, which equalled to reads of the sample with the second lowest number of reads. Rarefying eliminated thus one sample (a male, winter 2015, 1779 reads) from the data set. Thereafter, 598 taxa were left divided over 54 samples (for females six samples per season, and for males seven samples in summer 2013 and winter 2015, and eight samples in winter 2014 and summer 2014). The percentage of singletons increased after rarefying to 37.6%, while 13.5% of the ASVs were doubletons. The percentage of very low prevalence ASVs remained comparable to before rarefying, with 71.2% of the ASVs occurring in one sample.

#### Alpha-diversity

Similar to the host parameters, we identified correlations between alpha-diversity indices (richness, Shannon index, and Faith’s phylogenetic diversity) and season (summer vs. winter), sex, their interaction term (fixed factors) using linear mixed models with BirdID nested within aviary as random factors. We used again a stepwise backward exclusion of nonsignificant fixed factors and checked at each step of the analysis the normality of the model and homoskedasticity of the residuals. For the final model, we tested if aviary as a random factor contributed significantly (ANOVA). Using the same procedure, we next tested if the alpha-diversity indices were correlated with temperature or day length-related host characteristics in separate LMMs. To test temperature-related mechanisms, we used LMMs with metabolism indices (BMR and body mass) as fixed factors, and individual bird colour bands (BirdID) nested within aviary (random factors). Food intake and digestive efficiency were not included because they did not show seasonal variation (see Results) and because the data was limited to one year. To test day length-related mechanisms, we used LMMs with the seven innate immune indices as fixed factors, and individual bird colour bands (BirdID) nested within aviary (random factors). In almost all final models, aviary did not contribute significantly and was thus not included in the final models unless stated otherwise.

#### Community composition differences

The bacterial community composition (beta-diversity) was assessed by looking at the taxonomic similarities between seasons (summer vs winter) and sexes using the Jaccard similarity index (community membership: presence/absence), Bray–Curtis dissimilarities (community structure: presence/absence and abundance matrix), and by looking at the phylogenetic similarities between seasons and sexes using unweighted (community membership: presence/absence table) and weighted UniFrac distances (community structure: presence/absence/abundance matrix [62]). A principal coordinate ordination analysis (PCoA) of the beta-diversity indices was performed to test if community clustering and group dispersion differed between seasons or sexes, which was achieved by modelling beta-diversity (dis)similarities and distances from an ASV-level table using PERMANOVA with 10,000 permutations (adonis2 function in *vegan*) [63, 64]. Since we had multiple samples per individual, we first evaluated the effect of individuals on the different beta-diversity indices; this effect was always significant (*P* < 0.01). We next tested for the effect of season, sex, and their interaction while including individual as a blocking factor (*strata*) to control for the repeated sampling. We evaluated the degree of within-group dispersions (permutest) using the ‘betadisper’ function [65] in *vegan*. These were always nonsignificant, indicating that differences found were not due to differences in group dispersions.

For the three beta-diversity indices that showed seasonal differences, we tested if their ordination was comparable to the ordination of the metabolism indices (BMR and body mass) or the immune indices by performing a Procrustes analysis using the *Procrustes* and *Protest* functions in *vegan* [63, 64]. We analysed the similarity of the two-dimensional shapes produced from overlaying the principal component analyses of the Euclidian distances of metabolism or immune competence with the beta-diversity measure.

#### Taxonomic composition

We used the same LMM procedure as described above for the alpha-diversity indices to examine variation in the relative abundances of the most abundant phyla (> 5%) and genera (> 5%). As explanatory variables, we included season, sex, and their interaction term, metabolism (BMR and body mass), or immune competence (seven innate immune indices). Before running the LMMs, taxa proportions were logit transformed as log[(*p* + *e*)/(1 − *p* + *e*)], where *p* is the proportion of a taxon in a given sample and *e* the lowest proportion (among samples) for that taxon excluding zero [66].

#### Seasonal bacterial associates

To pinpoint which taxa may play a role in seasonal acclimatization in homing pigeons, we identified seasonal bacterial associates (i.e., core biomarkers [67]), that represent the bacterial ASVs whose ecology or function is likely important for the seasonal acclimatization of the host [68]. We determined the seasonal bacterial associates via two methods. First, we characterized seasonal bacterial associates as the ASVs that were more abundant in a season via a linear discriminant analysis (LDA) effect size analysis (LEfSe, [67]) on the online Huttenhower platform https://huttenhower.sph.harvard.edu/galaxy/), using the default settings. Since the Silva v132 database characterizes ASVs only at the genus level, we assigned a unique number to each ASV at the species level before performing the LEfSe analysis. The analysis was done for each sex separately, as sex significantly affected the indices of alpha- and beta-diversity.

Second, we characterized bacterial associates per season and per sex based on prevalence by comparing the core bacterial communities of a season or a sex with the overall core for all samples. ASVs were considered belonging to the core bacterial community when present in 90% of the samples a group (*microbiome* v1.10.0 [69]). Hence, when all samples are taken into account, core ASVs should occur in at least 49 of the 54 samples. Core-based bacterial associates for a season or sex where those ASVs that were unique for a season or sex when comparing their core ASVs with the core ASVs of all samples.

#### Functional profile of gut bacterial community

Lastly, we explored if the functional profile of the gut bacterial community showed seasonal differences. We used PICRUSt2 (version 2.3.0b) to predict KEGG ortholog (KO) metagenome functions from the 16S rRNA gene data using the rarefied data set [70, 71]. Note that although PICRUSt2 uses a larger and updated database than the original PICRUSt, the amplicon-based functional predictions are still limited by this reference database [64]. Functions of ASVs not included in the database may also be of importance to seasonal acclimatization of the host. We tested with PERMANOVA if KO function abundances varied between summer and winter, and between males and females, following the same approach as for the beta-diversity indices. Individual was again significant (*P* < 0.01) and included as blocking factor to control for repeated sampling (*strata*). Next, we identified which KO functions were more abundant in summer and which were more abundant in winter using a LEfSe analysis [67] for each sex separately.

## Results

### Seasonal variation in host parameters

As is common among birds living in temperate areas, pigeon body mass was higher in winter than in summer (Table 1; LMM, season *Ft*_1,38_ = 4.02, *P* = 0.05, sex *F*_1,12_ = 0.88, *P* = 0.37, season*sex *F*_1,38_ = 43.26, *P* < 0.001). Although the birds were heavier in winter, their total daily food intake did not differ between winter and summer, nor between sexes (LMM, season *F*_1,7_ = 0.022, *P* = 0.89, sex *F*_1,11_ = 0.003, *P* = 0.96, season*sex *F*_*1,7*_ = 0.111, *P* = 0.75). The digestive efficiency also did not vary with season or sex (LMM, season *F*_1,9_ = 0.34, *P* = 0.34, sex *F*_1,4_ = 1.56, *P* = 0.28, season*sex *F*_1,9_ = 0.06, *P* = 0.81). Consumption of different food components (pellets or seeds) did not differ between seasons (percentage pellets eaten; LLM initial model, season*sex *F*_1,7_ = 0.30, *P* = 0.60, season *F*_1,7_ = 0.33, *P* = 0.59). However, the average percentage pellets eaten by females (80.9%) was 1.8 times higher than the average percentage pellets eaten by males (46.0%; LMM final model, sex *F*_1,12_ = 7.85, *P* = 0.02). The sexual variation in food preference had no implications for the energy intake because the energy content did not differ between pellets and seeds (pellets: 17.47 kJ∙g^−1^, corn: 18.11 kJ∙g^−1^, peas: 17.89 kJ∙g^−1^, wheat: 18.00 kJ∙g^−1^). Hence all pigeons consumed the same amount of food and energy in summer and winter, and this did not differ between the sexes despite the sexual differences in diet preferences.

In general, BMR was higher in winter than in summer (Table 1; LMM, season *F*_1,35_ = 2.53, *P* = 0.12, sex *F*_1,12_ = 1.99, *P* = 0.18, season*sex *F*_1,35_ = 17.37, *P* < 0.01). In both sexes, BMR was lower in winter 2015 than in winter 2014 (but in males, in both winters BMR was higher than either summer), despite similar winter temperatures (4.0 °C in winter 2014 and 3.5 °C in winter 2015, Table 1).

Titres of antibodies against phosphorylcholine conjugated to BSA (i.e., anti-PC-BSA) were higher in summer than in winter (Table 1; LMM, season *F*_1,35_ = 0.63, *P* = 0.43, sex *F*_1,12_ = 0.42, *P* = 0.53, season*sex *F*_1,35_ = 4.18, *P* = 0.05). Haptoglobin concentrations (residuals after correcting for redness) varied with season and season*sex (Table 1; LMM, season *F*_1,36_ = 4.42, *P* = 0.04, sex *F*_1,12_ = 0.43, *P* = 0.53, season*sex *F*_1,36_ = 5.47, *P* = 0.03). Haptoglobin concentrations corrected for redness were higher in females, and in females also higher in winter than in summer. The five other immune indices did not vary with sex, and unexpectedly, also did not vary with season or season*sex (LMM, all *P* > 0.05; see Additional File 1: Table S2).

### Alpha-diversity

**Fig. 3:**
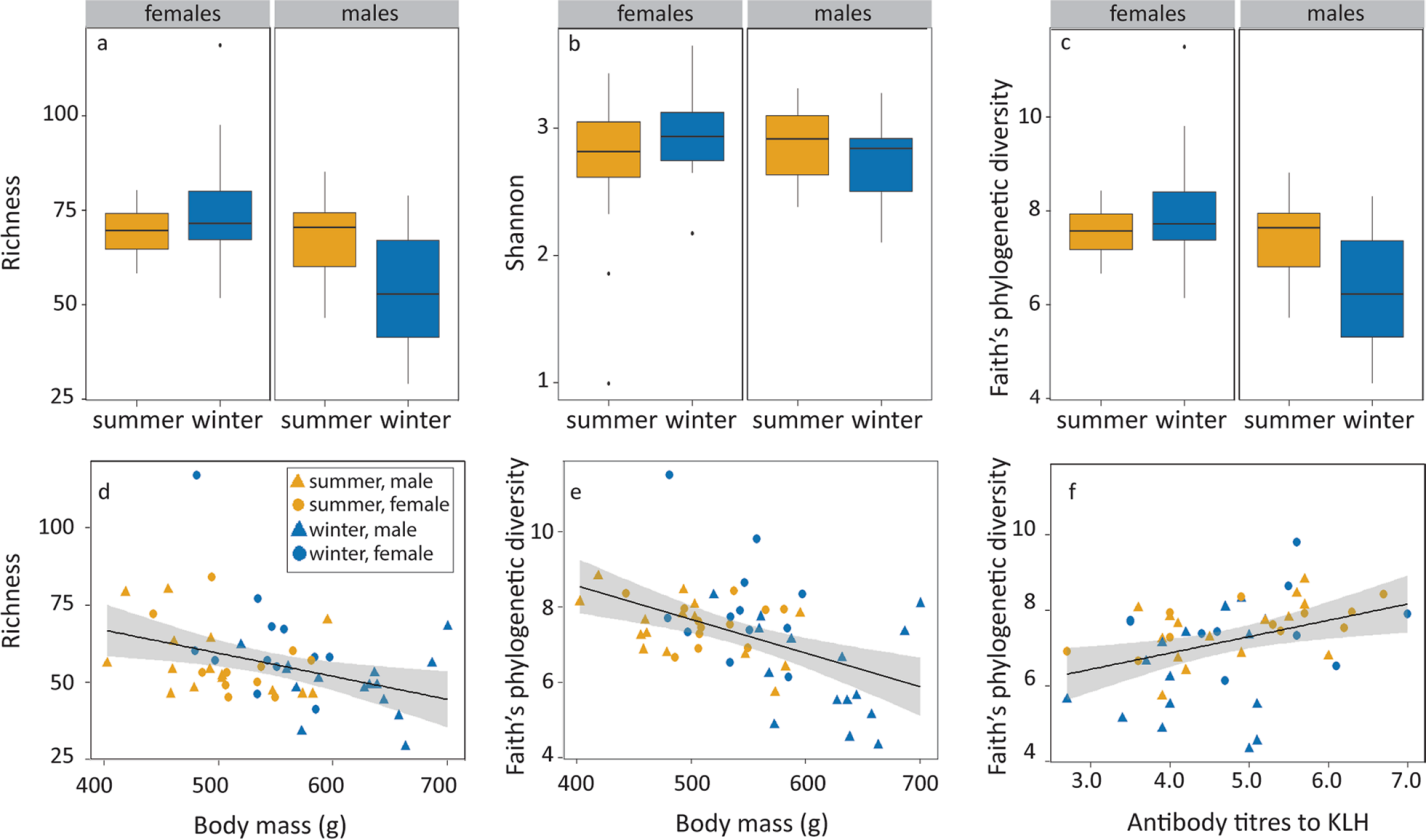
Relationships between alpha-diversity indices and season and sex, and metabolism and immune indices. The boxplots present seasonal and sexual variation for **a** richness, **b** Shannon diversity, and **c** Faith’s phylogenetic diversity. Richness and Faith’s phylogenetic diversity both decreased with increasing body mass **d**, **e**, while Faith’s phylogenetic diversity also increased with titres of antibodies against KLH **f**. Statistics are presented in Table 2

Richness was higher in females in winter than in summer but did not differ between seasons in males (season*sex *F*_1,38_ = 4.35, *P* = 0.04; Table 2, Fig. 3a). In addition, richness was negatively correlated with body mass (Fig. 3d) but was not correlated with any immune index. Shannon diversity did not differ between summer and winter or sexes (Fig. 3b), nor was it correlated with any metabolism or immune index. Faith’s phylogenetic diversity was lower in males in winter but did not differ between seasons in females (season*sex *F*_1,38_ = 9.09, *P* = 0.01; Table 2, Fig. 3c). Similar to the richness, Faith’s phylogenetic diversity was negatively correlated with body mass, but in addition, it was positively correlated with antibody titres against KLH (Fig. 3d, e).

**Table 2:** LMM analysis of the relationships between alpha diversities and season, sex, and the metabolic variables^a^

*Alpha diversity indices*	*Predictors final model*	*Df*	*F*	*P*
*Richness*				
Vs season, sex, season*sex	Season	1.38	1.86	0.18
	Sex	1.12	0.04	0.84
	Season*Sex	1.38	4.35	0.04
Vs BMR and body mass	Body Mass	1.39	7.92	0.01
*Faith’s PD*				
Vs season, sex, season*sex	Season	1.38	1.36	0.25
	Sex	1.12	0.08	0.78
	Season*Sex	1.38	9.09	0.01
Vs BMR and body mass	Body mass	1.39	14.22	< 0.01
Vs immune indices	KLH	1.36	7.21	0.01

^a^Only the fixed factors of the final models are presented. Faith’s PD is Faith’s phylogenetic diversity. Units of the factors: season, summer and winter; sex, female and male; BMR, ml O_2_∙h^−1^; body mass, g; and KLH, antibody titre against immune index KLH

### Community composition differences

**Fig. 4:**
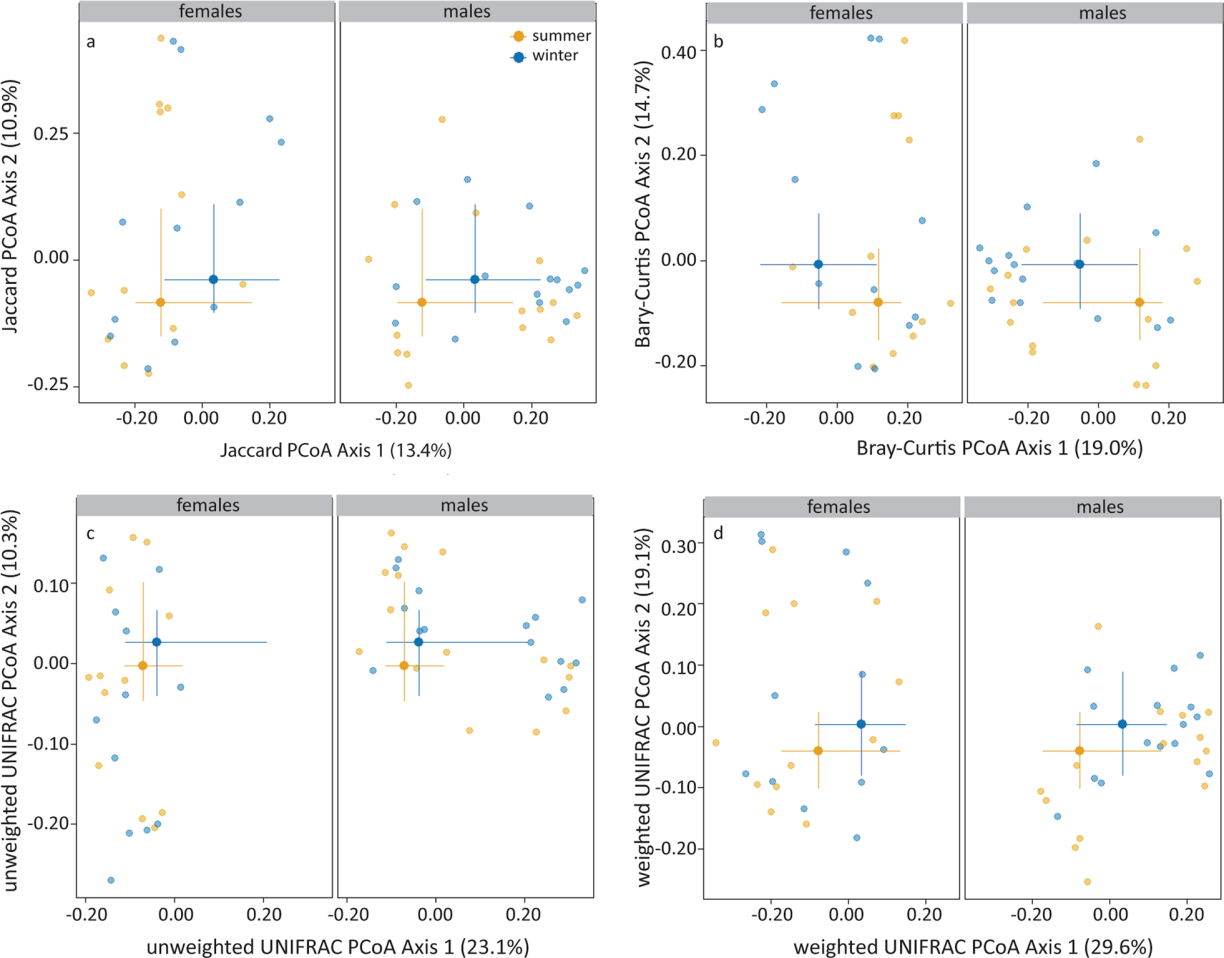
Seasonal and sexual variation in Jaccard (**a**) and Bray–Curtis (dis)similarities (**b**), and unweighted (**c**) and weighted UniFrac distances (**d**) depicted in PCoA plots. Statistics are presented in the text. The large symbols represent the medians, the error bars the 25% and 75% quantiles. The transparent symbols present the underlying data

Both taxon presence/absence (Jaccard, Bray–Curtis; Fig. 4a, b) and phylogenetic (weighted UniFrac, Fig. 4d) community composition varied with season and sex, but not their interaction term (Table 3). Unweighted UniFrac did not vary with season or sex (Fig. 4c). Season explained less of the variation in community composition (2.6–3.1%) than sex (6.5–12.8%). The ordination of the Jaccard and Bray–Curtis (dis)similarities, and weighted UniFrac distances matched with that of the metabolism indices (BMR and body mass; all Procrustes SS = 0.88, *P* = 0.01, Additional file 1: Fig. S2), but not with that of the immune indices (*P* = 0.96, *P* = 0.99, *P* = 0.73, for Jaccard, Bray–Curtis and weighted UniFrac, respectively).

**Table 3:** The permanova analyses of the relationships between beta diversity indices and season and sex^a^

*Beta diversity indices*	*Predictors final model*	*R*^*2*^* (%)*	*F*	*P*
Jaccard	Season	2.6	1.46	< 0.01
	Sex	6.5	3.65	< 0.01
Bray–Curtis	Season	3.1	1.8	< 0.01
	Sex	8.4	4.81	< 0.01
Weighted UniFrac	Season	2.6	1.55	0.01
	Sex	12.8	7.71	0.01

^a^Only the final models are presented. Units of the factors: season, summer and winter; sex, female and male

### Taxonomic composition

**Fig. 5:**
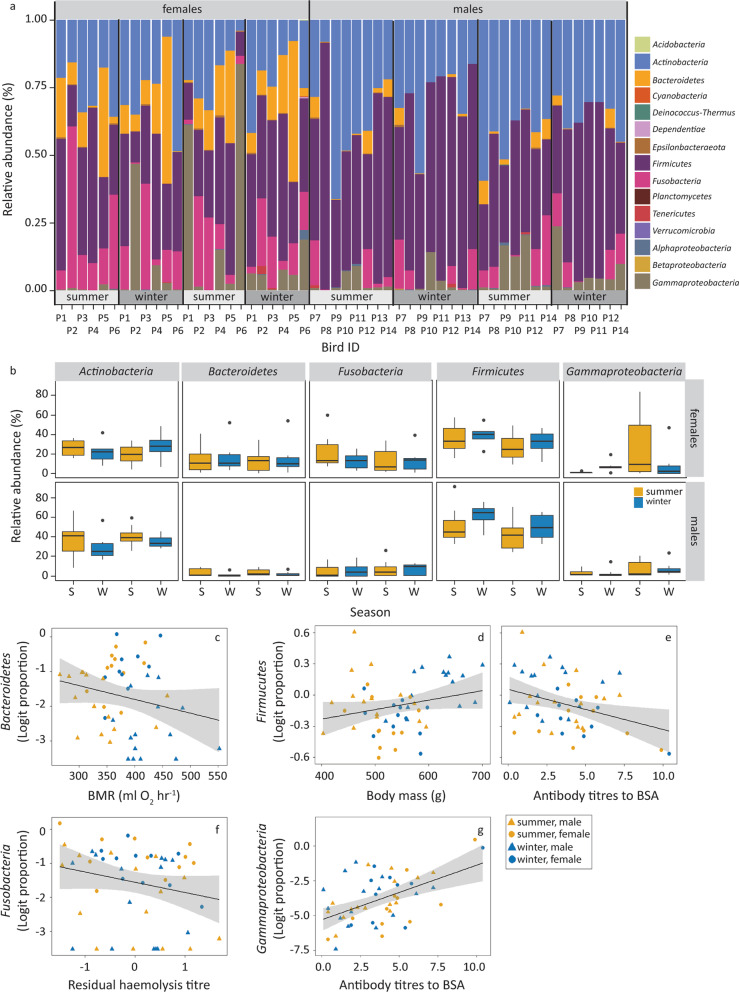
The variation in phylum relative abundance with season, sex, and immune indices. **a** Stacked bar plots of phylum relative abundance per sample. Note that the large phylum *Proteobacteria* was divided into the three classes present. **b** Boxplots of the relative abundances of the five most abundant phyla. The data is organized per sex and season (S = summer, W = winter), starting with the summer of 2013. The relationships between the logit(proportion) of *Bacteroidetes* and BMR **c**, the logit(proportion) of *Firmicutes* and body mass **d** and BSA titre **e**, the logit(proportion) of *Fusobacteria* and residuals of the haemolysis titre **f**, and the logit (proportion) of *Gammaproteobacteria* and BSA titre **g** are presented in separate panels. Statistics are presented in Table 4

The taxa were divided over 14 phyla, including an unclassified phylum belonging to an unclassified kingdom. We kept the latter in the analyses as we cannot exclude it were bacteria. For the subsequent analyses, we divided the large *Proteobacteria* phylum into three classes: *Alphaproteobacteria, Betaproteobacteria,* and *Gammaproteobacteria.* Five taxa had high mean relative abundances (> 5%; Fig. 5): *Firmicutes* (43.1% ± 17.7 SD), *Actinobacteria* (30.2% ± 13.5 SD), *Fusobacteria* (10.3% ± 11.9 SD), *Bacteroidetes* (8.2% ± 12.3 SD), and *Gammaproteobacteria* (7.8% ± 15.3 SD). These taxa are commonly found in avian gut bacterial community, apart from *Fusobacteria* [72, 73]. The logit proportion of *Bacteroidetes* varied significantly with season, being highest in summer (LMM, *F*_1,38_ = 7.46, *P* < 0.01, Fig. 5b, Table 4), while the logit proportions of *Firmicutes* tended to be higher in winter (*F*_1,36_ = 3.63, *P* = 0.06); note that this was not significant. Logit proportions of the other taxa did not vary with season. Logit proportions of *Bacteroidetes*, *Firmicutes*, *Actinobacteria* and *Fusobacteria* varied with sex (Table 4, Fig. 4). In addition, we found some correlations with metabolism and immune indices. The logit proportion of *Bacteroidetes* decreased with BMR (*F*_1,36_ = 9.81, *P* < 0.01, Fig. 5c). The logit proportion of *Firmicutes* increased with body mass and decreased with antibody titres against BSA (*F*_1,39_ = 4.30, *P* = 0.04 and *F*_1,36_ = 5.43, *P* = 0.03, respectively, Fig. 5d, e). The logit proportion of *Fusobacteria* decreased with haemolysis titres corrected for sample age (*F*_1,34_ = 4.96, *P* = 0.03, Fig. 5f). Lastly, logit proportions of *Gammaproteobacteria* increased with antibody titres against BSA (*F*_1,36_ = 14.73, *P* < 0.01).

**Table 4:** LMM analysis of the logit proportions of the most abundant phyla^a^ and genera^b^

*Taxa and model*	*Predictors final model*	*Df*	*F*	*P*
*Actinobacteria (p)*				
vs season, sex and season*sex	Sex	1,12	7.4	0.02
*Bacteroidetes (p)*				
vs season, sex and season*sex	Season	1,38	7.46	0.01
	Sex	1,12	10.06	0.01
	Season*Sex	1,38	16.84	< 0.01
vs BMR and body mass	BMR	1,36	9.81	< 0.01
*Firmicutes (p)*				
vs season, sex and season*sex	Sex	1,12	11.92	< 0.01
vs BMR and body mass	Body mass	1,39	4.3	0.04
vs immune indices	BSA	1,36	5.43	0.03
*Fusobacteria (p)*				
vs season, sex and season*sex	Sex	1,11	10.51	0.01
vs immune indices	Residual haemolysis	1,34	4.96	0.03
*Gammaprotecobacteria* (c)				
vs immune indices	BSA	1,36	14.73	< 0.01
*Actinomyces* (g)				
vs season, sex and season*sex	Season	1,39	4.14	0.05
*Bacteroides* (g)				
vs season, sex and season*sex	Season	1,38	0.15	0.7
	Sex	1,12	6.26	0.03
	Season*Sex	1,38	7.88	0.01
vs BMR and body mass	BMR	1,36	5.39	0.03
*Fusobacterium (g)*				
vs season, sex and season*sex	Sex	1,11^c^	5.23^c^	0.04^c^
*Lachnoclostridium* 12 (g)				
vs season, sex and season*sex	Season	1,39	15.26	< 0.01
vs BMR and body mass	BMR	1,35	8.08	0.01
	Body mass	1,35	8.01	0.01
*Oceanivirga* (g)				
vs season, sex and season*sex	Sex	1,11^c^	11.00^c^	0.01^c^
vs immune indices	Residual haemolysis	1,34^c^	4.47^c^	0.04^c^
Uncultured *Coriobacteriales* (g)				
vs season, sex and season*sex	Sex	1,12	9.29	0.01
vs immune indices	Residual haptoglobin	1,34 ^c^	5.81 ^c^	0.02 ^c^

^a^The *Proteobacteria* were divided in the classes present, its class *Gammaproteobacteria* belonged to the most abundant phyla and *Proteobacteria* classes. ^b^Only the fixed factors of the final models are presented. ^c^Model with aviary included as random factor. Units of factors: season, summer and winter; sex, female and male; BMR, ml O_2_∙h^−1^; body mass, g; and BSA, antibody titre against immune index BSA; residual haemolysis, lysis; and residual haptoglobin concentration, mg ml^−1^

Eight of the 128 genera present had high relative abundances (> 5%). The most abundant genus was *Lachnoclostridium* 12 (*Firmicutes*; 12.8% ± 13.2 SD), which included the most abundant ASV that was present in all samples (ASV nr. 093e1dd8072a68e5fa46226677183da). The logit proportion of *Lachnoclostridium* 12 was higher in winter than in summer (*F*_1,39_ = 15.26, *P* < 0.01, Additional file 1: Fig. S3 and Table 4). Logit proportions of two of the other high abundance genera also showed variation with season, both higher in summer: *Actinomyces* (10.8% ± 6.0 SD, *Actinobacteria*; season *F*_1,39_ = 4.14, *P* = 0.05), and *Bacteroides* (5.6% ± 6.6 SD, *Bacteroidetes*; season*sex *F*_1,38_ = 5.39, *P* = 0.01). Logit proportions of four genera varied with sex: logit proportions of *Bacteroides* (season*sex *F*_1,38_ = 5.39, *P* = 0.01), *Oceanivirga* (5.7% ± 7.7 SD; *F*_1,11_ = 11.00, *P* = 0.01, aviary contributed significantly to the model) and *Fusobacterium* (5.3% ± 5.8 SD; *F*_1,11_ = 5.23, *P* = 0.04) were higher in females, while the logit proportion of an uncultured *Coriobacteriales* (5.4% ± 3.3 SD) was higher in males (*F*_1,12_ = 9.29, *P* = 0.02). Logit proportions of two genera were correlated with metabolism indices: *Lachnoclostridium* 12 decreased slightly with BMR (*F*_1,35_ = 8.08, *P* < 0.01), and increased with body mass (*F*_*1,35*_ = 8.01, *P* < 0.01), while *Bacteroides* increased with BMR (*F*_1,36_ = 5.39, *P* = 0.03). Logit proportions of another two genera were correlated with immune indices: an uncultured *Coriobacteriales* increased with haptoglobin concentrations corrected for redness (*F*_1,34_ = 5.81, *P* = 0.03, aviary contributed significantly to the model), and *Oceanivirga* decreased with residual haemolysis corrected for sample age (*F*_1,34_ = 4.47, *P* = 0.04). Logit proportions of two genera did not vary with any factor: *Lactobacillus* (5.8% ± 12.4 SD, *Firmicutes*) and *Varibaculum* (7.7% ± 4.9 SD, *Actinobacteria*).

### Seasonal bacterial associates

**Fig. 6:**
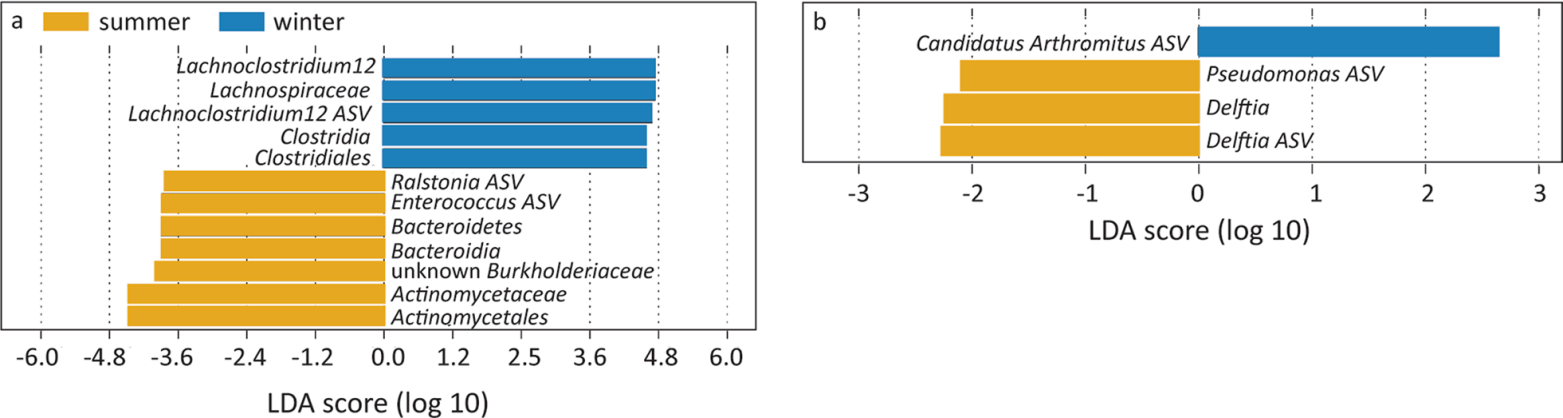
Seasonal LEfSe associates per sex, and the significant LMM results in individual associates. **a** Associates that were more abundant in summer (negative values) or winter ( − positive values) in male homing pigeons. **b** Associates that were more abundant in summer or winter in female homing pigeons

LDA effect size analysis (LEfSe) detected 12 seasonal bacterial associates in male homing pigeons, of which five were more abundant in winter and seven were more abundant in summer (Fig. 6a). The five winter LEfSe-based bacterial associates in males belonged all to the *Firmicutes*’ class *Clostridia* including the most abundant genus and ASV (*Lachnoclostridium* 12 ASV nr. 093e1dd8072a68e5fa46226677183da). The seven male summer LEfSe-based bacterial associates belonged to four phyla: *Firmicutes* (an *Enterococcus* ASV), *Actinobacteria* (*Actinomycetales* and *Actinomycetaceae*), *Bacteroidetes* (*Bacteroidetes* itself and *Bacteroidia*), and *Proteobacteria* (*Burkholderiaceae* and a *Ralstonia* ASV). In females, four seasonal bacterial associates were present. The winter bacterial associate belonged just as in males to the *Clostridia* (*Firmicutes*; *Candidatus Arthromitus* ASV), the three summer bacterial associates belonged to the *Proteobacteria* (*Pseudomonas* ASV, *Delftia* ASV and the *Delftia* genus; Fig. 6b).

Next we determined seasonal bacterial associates based on prevalence, by comparing the summer and winter core ASVs with the overall core ASVs. The overall core bacterial community (ASVs occurring in 90% of all samples) consisted of 13 ASVs, of which eight belonged to the *Actinobacteria* phylum and five to the *Firmicutes* phylum (Table 5). As expected, the three most abundant ASVs belonged to the overall core bacterial community: *Lachnoclostridium* 12 ASV (*Firmicutes*), an *Actinomyces* ASV (10.8%) and *Varibaculum* ASV (7.7%, both *Actinobacteria*). These ASVs occurred in all samples, as did a *Negativicoccus* ASV (4.1%, *Firmicutes*). The other core ASVs had intermediate abundances, but two core ASVs had low relative abundances: *Varibaculum* ASV (0.6%, *Actinobacteria*) and the unclassified *Propionibacteriaceae* ASV (0.3%, *Actinobacteria*).

**Table 5:** Core ASVs for all (all), summer (S), winter (W), female (F) or male (M) samples

*Phylum*	*Class*	*Order*	*Family*	*Genus*	*ASV nr*	*All*	*S*	*W*	*F*	*M*
*Actinobacteria*	*Actinobacteria*	*Actinomycetales*	*Actinomycetaceae*	*Actinomyces*	92a2c02d4eb50b74d759e8768f35de75	√	√	√	√	√
					1dabaea4df2042917cab9756bd51f88f	√	√		√	√
				*Uncultured*	299cf49884b81d42c2f9bfcbbd193416	√	√	√	√	√
				*Varibaculum*	385a6763190dc35cc99ea7eca5148740	√	√	√	√	√
					336ead086d83a1206a97ac8e76ee59ea	√	√		√	√
		*Corynebacteriales*	*Corynebacterium*	*Corynebacterium1*	d7141f31a41d78e1bea19258d3193f50			√		
				*Lawsonella*	41abb52daaf5f9b3401da0572950c0a8					√
		*Propionibacteriales*	*Propionibacteriaceae*	*Unknown*	2a65734a05731c512e9cf9d846840525	√	√			√
	*Coriobacteriia*	*Coriobacteriales*	*Atopobiaceae*	*Atopobium*	135b33c0c8bdf13e5b2c2582eaa8367c	√	√	√	√	√
			*uncultered*	*Unclassified*	9c745d4b489d8cd43869b697ff740930	√	√	√	√	√
*Firmicutes*	*Bacilli*	*Lactobacilales*	*Lactobacillalceae*	*Lactobacillus*	463b54affc2cffb39f5f06feac1a5866			√		
	*Clostridia*	*Clostridiales*	*Family XI*	*Anaerococcus*	740503fa229c763d76186ffa0cb1689a			√		
				*Murdochiella*	992ced6c431c713b6d0c7dd342acafd2	√	√	√	√	√
				*Peptoniphilus*	b65c9ee376814eca8ffdfb8e320c3af8	√	√	√	√	√
			*Lachnospiraceae*	*Lacnoclostridium 12*	7093e1dd8072a68e5fa46226677183da	√	√	√	√	√
			*Peptococcaceae*	*Peptococcus*	559f896fc6eafd23e47b0eacbe3e7b04				√	
			*Ruminococcaceae*	*Fastidiosipila*	2fb6cf1197894ee035ca9b023dbba394				√	
				*Unclassified*	7372498c13452d74921bd9b4faed2d37	√	√		√	√
	*Negativicutes*	*Selenomonadales*	*Veillonellaceae*	*Negativicoccus*	7dbdd4cda4df635e17d5ad7a47aca593	√	√	√	√	√
*Fusobacteria*	*Fusobacteriia*	*Fusobacteriales*	*Fusobacteriaceae*	*Fusobacterium*	4f8d3dc2be35252dd3f1aff6ba4a49f2				√	
			*Leptotrichiaceae*	*Oceanivirga*	2e47ceb43f8943a5bef712c18b54733b				√	

The summer core bacterial community consisted of the same 13 ASVs as the overall core bacterial community (Table 5), and thus we could not detect summer-specific bacterial associates based on prevalence. However, three of the 12 ASVs of the winter core bacterial community were unique to the winter core: a *Corynebacterium 1* ASV (*Actinobacteria*), and a *Lactobacillus* and *Anaerococcus* ASV (both *Firmicutes*, Table 5). Four of the overall core ASVs were not present in the winter core bacterial community: an *Actinomyces*, a *Varibaculum*, and the unclassified *Propionibacteriaceae* ASV (*Actinobacteria*), and an unclassified *Ruminococcaceae* ASV (*Firmicutes*).

We also determined core-based bacterial associates for each sex. The male core bacterial community (14 ASVs, Table 5) included the overall core bacterial community plus one unique ASV, a *Lawsonella* ASV (*Actinobacteria*). The female core bacterial community (16 ASVs) included four unique ASVs: a *Fastidiosipila* and a *Peptococcus* ASV (both *Firmicutes*), and *Oceanivirga* and a *Fusobacterium* ASV (both *Fusobacteria*, a phylum not present in the overall core bacterial community). One overall core bacterial community did not occur in the female core microbiome: the unclassified *Propionibacteriaceae* ASV (*Actinobacteria*) that was also not present in the winter core bacterial community.

### Functional profile

**Fig. 7:**
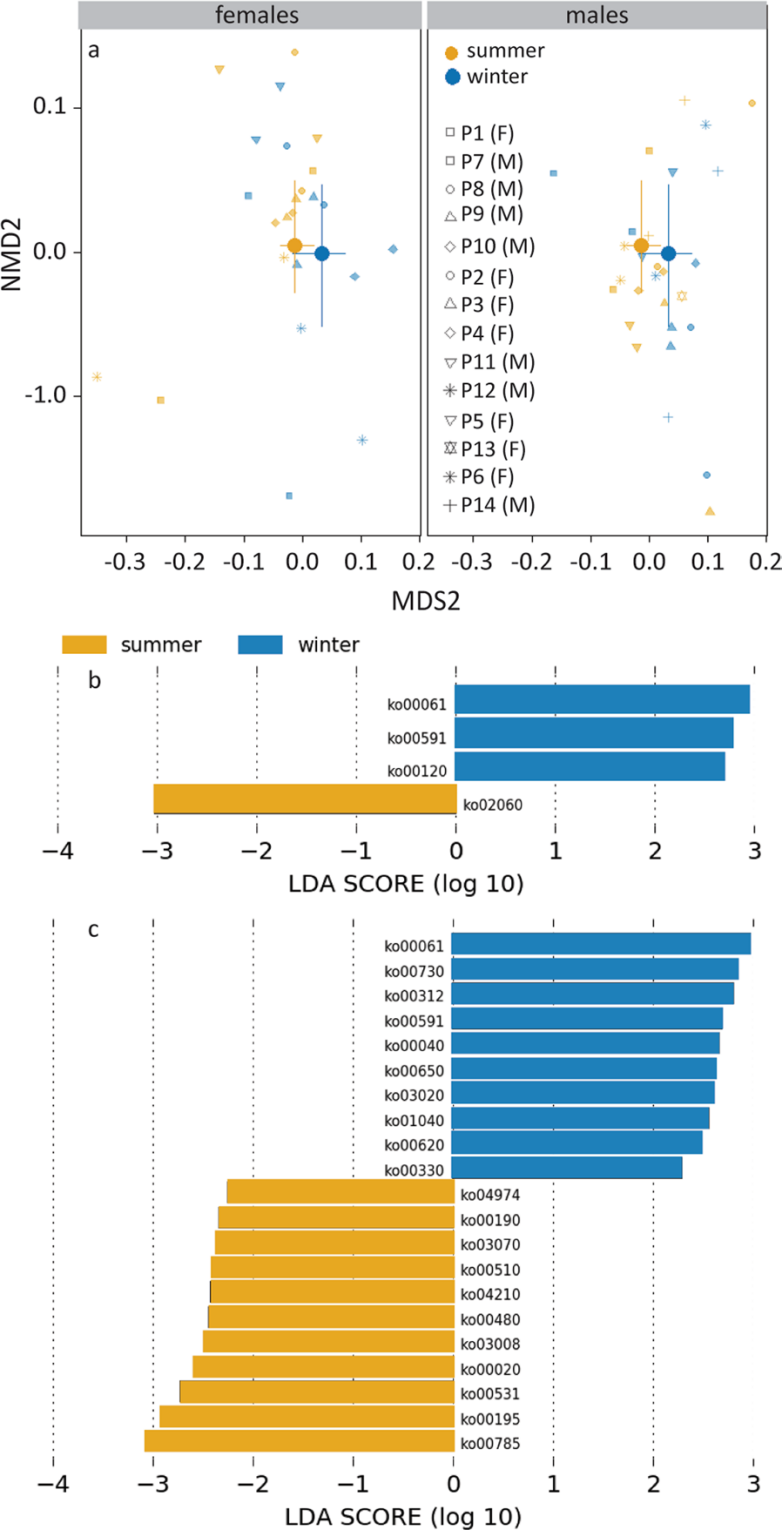
Seasonal and sexual variation in KO pathway abundances. **a** MDS plot of seasonal differences within sex. **b** KO pathways that were more abundant in summer or winter in females. **c** KO pathways that were abundant in summer or winter in males. Statistics are presented in the text. The large symbols represent the medians, the error bars the 25% and 75% quantiles. The transparent symbols present the underlying data for the individual pigeons

The PICRUSt2 analysis yielded 161 KO metagenome functions in total. The KO function abundances differed by season (PERMANOVA, *R*^2^ = 0.035, *F*_1,53_ = 2.00, *P* = 0.03) and sex (*R*^2^ = 0.075, *F*_1,53_ = 4.30, *P* = 0.03; Fig. 7a). The season*sex interaction was not significant. In females, three KO functions were more abundant in winter, and one KO function was more abundant in summer (LEfSe analyses; Fig. 7b). In males, 10 KO functions were more abundant in winter, and 11 KO functions were more abundant in summer (Fig. 7c). Males and females shared two KO functions that were more abundant in winter, while none of the summer-specific KO functions were shared. The shared winter-specific KO functions are important to metabolism and lipid metabolism: fatty acid biosynthesis (KO00061) and linoleic acid metabolism (KO00591). In addition, in males another winter-specific KO function is important to metabolism and lipid metabolism: KO01040 (biosynthesis of unsaturated fatty acids). In females, the third winter-specific KO function (KO00120, primary bile acid biosynthesis), may be involved in the response of gut bacterial community to day length differences [32].

Please note, that given the limitations of the PICRUSt2 analyses also other KO functions may be important for seasonal acclimatization in pigeons.

## Discussion

We examined if other factors than diet, such as temperature and day length, play a role in shaping seasonal variation in the gut bacterial community in birds. Specifically, we investigated whether the gut bacterial community of homing pigeons that lived outdoors differed between summer and winter despite a constant diet. We tested whether seasonal variation in the gut bacterial community was correlated with host metabolism, immune function or both. Metabolism is a potential intermediary between seasonal changes in temperature and the animal’s gut bacterial community. Similarly, immune competence might link seasonal changes in day length and the gut bacterial community. All the characteristics of the gut bacterial community showed summer–winter differences (expectation 1). Temperature likely contributed to the summer–winter differences in the gut bacterial community, as the relative abundances of *Firmicutes* tended to be higher in winter and relative abundances of *Bacteroidetes* were higher in summer (expectation 2), and multiple gut bacterial community characteristics were correlated with at least one metabolism index (expectation 3). Lastly, we found correlations between immune indices and gut microbiota characteristics (expectation 4).

In addition to summer–winter differences, most gut bacterial community characteristics differed between males and females. These sex difference may have been driven by corresponding differences in diet. For example, we documented that the percentage pellets in the diet was 1.8 times higher in females than in males. However, the pellets and seed mixture did not differ much in terms of nutrition (i.e., crude protein, crude fat, crude fibre, crude ash contents, and energy content; Additional file 1: Table S1). Instead, sex-specific physiological mechanisms may have played a more important role in structuring gut microbiota within each sex, but the nature and consequences of such mechanisms require further investigation.

### Seasonal temperature variation contributed to seasonal gut bacterial community variation

A strong indication that seasonal temperature differences partly caused the summer–winter differences in the gut bacterial community was the tendency of the relative abundances of *Firmicutes*, a phylum previously associated with low temperature [20, 23], to peak in winter, and the many *Firmicutes* taxa present among the winter bacterial associates. The higher winter relative abundances of *Firmicutes* mainly resulted from changes in the *Clostridia* class, which contained the most abundant genus (*Lachnoclostridium* 12) and ASV (*Lachnoclostridium* 12 ASV). These *Lachnoclostridium* 12 taxa were LEfSe-based winter associates in males, and peaked over all samples in abundance in winter just as their higher taxa (*Lachnospiraceae*, *Clostridiales*, *Clostridia*). These taxa are thus important components of the winter gut bacterial community in homing pigeons. In humans, *Lachnospiraceae* are recognized as an essential part of the core bacterial community that promotes health [74]. *Lachnospiraceae* comprises anaerobic, fermentative, and chemoorganotrophic bacteria, that produce short-chain fatty acids (SCFAs) like butyrate by hydrolysing carbohydrates [74]. SCFAs fulfil vital functions in animals. They provide an energy source, maintain intestinal epithelium physiology, regulate innate and adaptive immune function, and may reduce inflammation [75–78], but SFCAs may also influence the regulation and capacity of energy regulation [79]. Of the two predicted KO functions that were more abundant in winter in both sexes, one (KO00061) represents fatty acid biosynthesis and the other (KO00591) linoleic acid metabolism. Considering the importance of SCFAs, their increased biosynthesis by the gut bacterial community may be especially beneficial. SCFAs produced by the gut bacterial community might even contribute to overwinter survival of hosts. In winter, energy budgets can come under pressure, for example, due to increased thermoregulation and foraging costs, but the gut bacterial community-produced SCFAs may alleviate some of this pressure. Enhanced bacterial metabolism of linoleic acid, a polyunsaturated omega-6 fatty acid (PUFA; 18:2*n*6), may also offer advantages. High-PUFA diets are beneficial to migrating birds because they reduce the energy expenditure during long-duration flights, which are otherwise energetically demanding [79, 80]. This benefit may be due to PUFAs increasing the amount of transport proteins and catabolic enzymes that deliver fatty acids to mitochondria [81]. Linoleic acid and other PUFAs may offer similar benefits to wintering birds facing increased energy expenditures.

**Fig. 8:**
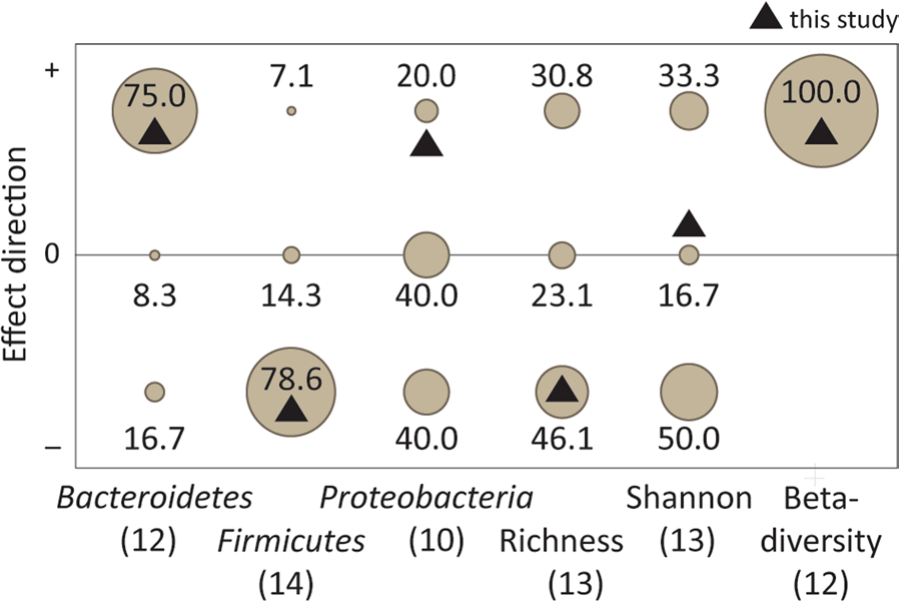
The effect directions of temperature on some aspects of vertebrate gut bacterial community found in 18 studies and 17 species. Literature sources and data, including some additional data, are presented in Additional file 1: Table S3. A positive effect direction ( +) indicates an increase with temperature or a significant difference in beta-diversity, a negative effect direction ( − ) indicates a decrease with temperature (does not apply to beta-diversity), and the 0 indicates no significant effect. Circle size represents the percentage of studies with that effect direction within the studies that reported on the concerned index; this percentage is specified by the numbers near the circles. The number of studies that presented data for the given taxa, alpha- and beta-diversity indices are indicated between brackets under the X-axe labels

Our results showed agreements and disagreements with 18 published studies on the effects of temperature on the gut bacterial community in vertebrates (mammals, birds, reptiles, amphibians, and fish; Fig. 8 and Additional file 1: Table S3). Most of these studies focused directly on temperature effects (14 lab studies), but some focussed on seasonal effects (one husbandry and three field studies). In a majority of the studies, including ours, relative abundances of *Firmicutes* were highest at lower temperatures and relative abundances of *Bacteroidetes* were highest at higher temperatures. *Bacteroidetes* also ferment carbohydrates and produce SCFAs [82]. The alternating peaks in relative abundances of *Firmicutes* and *Bacteroidetes* between seasons, suggests that differences in the carbohydrate fermentation products between these taxa may play a role in seasonal acclimatization in homing pigeons and other vertebrates. The alternating peak also lead to a higher ratio of *Firmicutes*:*Bacteroidetes* in winter, which was most evident in males (LMM, season*sex *F*_1,31_ = 4.79, *P* = 0.04; Additional file 1: Fig S4). In mammals, the cold-associated increase in the *Firmicutes*:*Bacteroidetes* ratio is associated with aspects of cold acclimatization in host metabolism. The higher ratio is associated with enhanced energy extraction and thus increased energy consumption [27]; it is also associated with high-fat diets [83] and obesity [84]. Additional body mass in winter, as we observed in our homing pigeons, is an adaptive trait in animals living in temperate or cold areas. Increased body reserves promote survival during the harsher winter period. A decrease in the *Firmicutes*:*Bacteroidetes* ratio at warmer temperatures is associated with fasting [71], and protection against obesity [84]. These affects, especially the latter, are beneficial in summer in wild animals. In addition, it is noteworthy that both *Firmicutes* and *Bacteroidetes* relative abundances were correlated with body mass or BMR, suggesting that also in homing pigeons, host metabolism may mediate the temperature effects on gut bacterial community. All in all, in homing pigeons the observed seasonal variation in the *Firmicutes*:*Bacteroidetes* ratio suggests that seasonal patterns in gut bacterial community may be attributed to acclimatization to seasonal temperature changes.

Many of the 18 studies also reported the effects of temperature on alpha-diversity indices (richness and Shannon index) and the relative abundances of *Proteobacteria* (Fig. 7). There was no general trend for these variables. Beta-diversity indices, on the other hand, always differed between temperatures or seasons when reported, similar to this study. Thus, our results closely match the general temperature effects on the gut bacterial community presented in the literature, indicating that the seasonal variation in environmental temperature contributed considerably to the summer–winter differences in the gut bacterial community of homing pigeons.

### Is immune competence a link between day length and the gut bacterial community?

The gut bacterial community is known to have strong and dynamic interactions with especially the innate immune system [76–78]. For instance, the SFCAs produced by the gut bacteria play an essential role in the host (intestinal) immune defence. These molecules interact with the intestinal epithelial cells, reduce intestinal inflammation, provide protection against pathogens, and regulate activation and differentiation of immune cells [76–78]. We found multiple correlations between the immune indices and the characteristics of the gut bacterial community, but not between immune indices and the beta diversity indices. However, these correlations did not reveal consistent involvement of one or more specific immune indices. This lack of consistency complicates interpretations. Moreover, in contrast to the metabolism indices, only two of the seven immune function indices (i.e., antibody titres to PC-BSA and haptoglobin concentration corrected for redness) showed seasonal variation. Of these, only the haptoglobin concentration was correlated with an aspect of the gut bacterial community (relative abundances of uncultured *Coriobacteriales*). Given this complexity of the results and lack of seasonal differences in the immune indices, our study does not clearly support the idea that innate immune function indices mediates the previously documented links between daylength and the gut bacterial community. The lack of clear correlations between the gut microbiota and the innate immune indices may also be due to the modest number of individuals included in the study (14 homing pigeons), because generally, and also here, there is a large individual variation in the host-associated bacterial community. Note that we nevertheless do find consistent and strong effects of temperature to the seasonal variation in the gut bacterial community.

## Conclusions

Seasonal environmental variation influenced the gut bacterial community in homing pigeons, even when the birds were fed a constant diet. Temperature likely drove part of the seasonal differences in the gut bacterial community composition because we found multiple correlations between the characteristics of the gut bacterial community and metabolism indices. Furthermore, the summer–winter differences in the characteristics of the gut bacterial community matched previously described effects of temperature variation on the vertebrate gut bacterial community. In addition, in winter, the *Firmicutes*:*Bacteroidetes* ratio was higher, and fatty acid related predicted KO functions were more abundant, indicating that the seasonal variation in the gut bacterial community contributes to seasonal acclimatization of the host. We found less consistent correlations between the gut bacterial community characteristics and innate immune indices, and we conclude conservatively that the here used innate immune competence may be an unlikely link between day length and the gut bacterial community. However, we do not exclude that day length may have contributed to the seasonal differences in the gut bacterial community. Overall, our results highlight the need for future studies that disentangle different seasonally-varying factors (i.e., temperature, daylength, behaviour, diet, etc.) if the goal is to fully understand the causal mechanisms driving seasonal variation in the gut bacterial community.

## Supplementary Information

**Additional file 1.** Supplementary information including methods, tables and figures.

## Data Availability

The datasets supporting the conclusions of this article are available in the DataverseNL repository, https://doi.org/10.34894/ULA6QL. These include the biom data, phylogenetic tree and metadata used to create the *Phyloseq* object that was analysed in the current study, and the R-script used.
