# Supplementary material for: Gut microbiota of homing pigeons shows summer–winter variation under constant diet indicating a substantial effect of temperature
Source: Anim Microbiome. 2022 Dec 13;4:64. doi: 10.1186/s42523-022-00216-6 (PMC9749179; doi:10.1186/s42523-022-00216-6)
Supplement: Supplementary file 1 — Additional file 1. Supplementary information including methods, tables and figures. [file 42523_2022_216_MOESM1_ESM.docx]

**Additional file to:**

**Gut microbiota of homing pigeons shows summer-winter variation under constant diet indicating a substantial effect of temperature**

Maurine W. Dietz^1,*^, Kevin D. Matson^2^, Maaike A. Versteegh^1^, Marco van der Velde^1^, Henk K. Parmentier^3^, Joop A.J Arts^3^, Joana F. Salles^1^, B. Irene Tieleman^1^

^1^ Groningen Institute for Evolutionary Life Sciences, University of Groningen, Nijenborgh 7, 9747AG Groningen, Netherlands.

^2^ Wildlife Ecology and Conservation, Environmental Science Group, Wageningen University, Droevendaalsesteeg 3a, 6708PB Wageningen, Netherlands

^3^Adaptation Physiology, Department of Animal Sciences, Wageningen University, De Elst 1, 6708WD Wageningen

*Corresponding author, e-mail: [M.W.Dietz@rug.nl](mailto:M.W.Dietz@rug.nl)

**Contents Page**

**Methods: Calculation of the antibody titres** 2

**Table S1** Compositions of the two food types offered to the homing pigeons 3

**Table S2** LMM analysis of the relationships between host parameters and season and sex 4

**Table S3** Overview of the KEGG pathways (KOs) specific for winter and summer per sex 5

**Table S3** Ambient temperature effects on the vertebrate gut bacterial community: phylum 6

relative abundance, alpha-diversity, and beta-diversity

**Fig. S1** Richness rarefaction curves 9

**Fig. S2** Results of the Procrustes analyses 9

**Fig. S3** Significant variation in the Logit-transformed proportions of the most abundant 10

genera with season, sex, metabolism or immune indices

**Fig S4** Seasonal variation in the ratio *Firmicutes*/*Bacteroidetes* proportions in (**a**) females 11

and (**b**) males.

**Methods: Calculation of the antibody titres**

Antibody titres were calculated as described by [1] (taken from [2]). Briefly, the optical densities (OD) of the duplicate standard positive plasma were averaged for each plate. Logit values of the OD per plate were calculated using:

*Logit OD = ln (OD/(OD_max_ – OD))*

Where OD is the OD of a well, and OD_max_ is the maximum averaged OD of the duplicate standard positive plasma samples. The last positive well (lpw) of the averaged duplicate standard positive plasma sample was set to the sixth dilution. A linear regression line of the logit OD against the respective log_2_-dilution values of the averaged duplicate standard positive plasma samples was determined, which resulted in a regression coefficient β. Titres of the plasma samples per plate were calculated using:

*Titre = (logit OD_lpw_ – (logit OD_sample_ – β x log_2_(dilution_sample_)))/ β*

Were logit OD_lpw_ is the estimated logit OD at the lpw calculated with the estimated linear regression function using the log_2_-dilution value of that well, logit OD_sample_ is the logit OD calculated of the OD closest to 50% of OD_max_ for a plasma sample of an individual (OD_sample_), β is the regression coefficient of the estimated linear regression function of the averaged duplicate standard positive plasma samples, and log_2_ (dilution_sample_) is the log_2_-dilution value at which OD_sample_ occurred.

**References**

1. Frankena K. Immunological Techniques. Interact between *Cooperia* spp *Ostertagia* spp (*Nematoda*, *Trichostrongylidae*) Cattle. PhD Thesis Wageningen: Agricultural University Wageningen; 1987.

2. de Koning DB, Damen EPCW, Nieuwland MGB, van Grevenhof EM, Hazeleger W, Kemp B, et al. Association of natural (auto-) antibodies in young gilts with osteochondrosis at slaughter. Livest Sci; 2015;176:152–60. https://doi.org/10.1016/j.livsci.2015.03.017.

**Table S1** Compositions of the two food types offered to the homing pigeons.

|  | ***4 seasons Kasper^TM^ 6705*** | ***Pellets Kasper^TM^ P40*** |
| --- | --- | --- |
| Crude protein  Crude fat  Crude fibre  Crude ash | 12.3%  3.0%  3.4%  1.8% | 15.5%  2.8%  2.2%  4.0% |
| Composition | Corn, wheat, sorghum, yellow peas, barley, dunpeas, green peas, white sorghum, safflower seed, peeled oats and soybean oil. | Corn, wheat, soybean meal feed, corn gluten, calcium carbonate, monocalcium phosphate, dextrose, sodium chloride, alfalfa, timothee grassmeal, grassmeal, sunflower seed extracted, dried beet pulp, cane molasses, rape seed extracted, linseed, linseed expeller, soybean oil, and added vitamins, minerals, and amino acids. |

**Table S2** LMM analysis of the relationships between host parameters and season and sex^a^.

| ***Host parameter**** | ***Predictors final or initial model^b^*** | ***Df*** | ***F*** | ***P*** |
| --- | --- | --- | --- | --- |
| Body mass  BMR  HP(res)  HL(res)  KLH  HuSA  BSA  PC-BSA  Total consumption  Pellet consumption  Digestive efficiency | Season  Sex  Season*Sex  Season  Sex  Season*Sex  Season  Sex  Season*Sex  Season  Sex  Season*Sex  Season  Sex  Season*Sex  Season  Sex  Season*Sex  Season  Sex  Season*Sex  Season  Sex  Season*Sex  Season  Sex  Season*Sex  Sex  Season  Sex  Season*Sex | 1,38  1,12  1,38  1,35  1,12  1.35  1,36  1,12  1,36  1,33  1,11  1,33  1,32  1,11  1,32  1,32  1,11  1,32  1,32  1,11  1,32  1,35  1,12  1,35  1,7  1,11  1,7  1,12  1,9  1,4  1,9 | 4.02  0.88  43.26  2.53  1.99  17.37  4.42  0.43  5.47  0.04  0.07  0.11  0.04  0.10  0.79  0.0003  0.19  0.05  0.03  1.63  0.26  0.63  0.42  4.18  0.02  0.003  0.11  7.85  1.02  1.56  0.06 | 0.05  0.37  <0.0001  0.12  0.18  <0.001  0.04  0.53  0.03  0.85  0.79  0.74  0.84  0.75  0.38  0.99  0.68  0.82  0.86  0.53  0.61  0.43  0.53  0.05  0.89  0.96  0.75  0.02  0.34  0.28  0.81 |

^a^Only the fixed factors of the final models are presented. Units of the factors: season, summer and winter; sex, female and male. For HL(res), KLH, HuSA, BSA, total consumption and the digestive efficiency, the initial model is presented, as these factors were not correlated with season nor sex. *Units of host parameters: body mass was determined when the cloacal swab was taken (g), BMR is basal metabolic rate (ml O_2_∙h^-1^); the innate immune indices are: HP(res), residual haptoglobin concentration (mg ml^-1^), HL(res), residual haemolysis, HG, hemagglutination, KLH, keyhole limpet hemocyanin, HuSA, human serum albumin, BSA, bovine serum albumin, and PCBSA, phosphorylcholine conjugated to BSA (unit latter six indices: antibody titres against the immune indices); total consumption: the total amount of food eaten (g); pellet consumption: the percentage pellets in the diet; and the digestive efficiency, i.e., the assimilation quotient. Sample sizes were 6 females (F) and 8 males (M), except for summer 2013 when we had data of 7 males.

**Table S3** Overview of KEGG ortholog (KO) functions specific for winter and summer per sex.

| ***Sex*** | ***KO winter*** | ***Description*** | ***KO summer*** | ***Description*** |
| --- | --- | --- | --- | --- |
| **Males** | KO00040  KO00061  KO00312  KO00330  KO00591  KO00620  KO00650  KO00730  KO01040  KO03020 | Pentose and glucuronate interconversions  Fatty acid biosynthesis  Beta-Lactam resistance  Arginine and proline metabolism  Linoleic acid metabolism  Pyruvate metabolism  Butanoate metabolism  Thiamine metabolism  Biosynthesis of unsaturated fatty acids  RNA polymerase | KO00020  KO00190  KO00195  KO00480  KO00510  KO00531  KO00785  KO03008  KO03070  KO04210  KO04974 | Citrate cycle (TCA cycle)  Oxidative phosphorylation  Photosynthesis  Glutathione metabolism  N-Glycan biosynthesis  Glycosaminoglycan degradation  Lipoic acid metabolism  Ribosome biogenesis in eukaryotes  Bacterial secretion system  Apoptosis  Protein digestion and absorption |
| **Females** | KO00061  KO00120  KO00591 | Fatty acid biosynthesis  Primary bile acid biosynthesis  Linoleic acid metabolism | KO02060 | Phosphotransferase system (PTS) |

**Table S3** Ambient temperature effects on the vertebrate gut bacterial community: phylum relative abundance, alpha-diversity, and beta-diversity.

| ***Species*** | ***Location*** | ***Cold exposure*** | ***Gut bacterial community effects ^a^*** | ***Source*** |
| --- | --- | --- | --- | --- |
| Mammals  Alpine musk deer (*Moschus chrysogaster*)  Brandt’s vole (*Lasiopodomys bandtii*)  Brandt’s vole (*Lasiopodomys bandtii*)  Forest musk deer (*Mochus berezovskii*)  Mouse C57BI/6J (*Mus musculus*)  Mouse C57BL6/J (*Mus musculus*)  Mouse wild-type (*Mus musculus*)  Siberian flying squirrel (*Pteromys volans orii*)  Wild blue sheep (*Pseudois nayaur*) | Breeding centre,at Xinglong Mountain. China  Lab  Lab  Breeding centre, at the Qinghai-Tibet plateau, China  Lab  Lab  Lab  Field: Hokkaido forest, Japan  Field: Helan Mountain, China | Natural temperatures in spring and winter  3 weeks at 4°C or 23°C  4 weeks at 4°C, 4 weeks at 4°C followed by 4 weeks at 23°C, or 4-8 weeks at 23°C  Natural temperatures in summer and winter    Up to 10 days at 6°C or room temperature  Up to 6 days at 12°, 17° or 23°C  6 days at 6°C or 30°C  May-August, 8°-22°C  Summer (~17°C) or winter (~-9°C) | *Firmicutes* ↘  *Bacteroidetes* ↗  Richness ↘  Shannon ↘  Beta-diversity +  *Firmicutes* *−*  *Bacteroidetes* ↗  *Proteobacteria* −  Faith’s PD −  Beta-diversity +  *Firmicutes* ↘  *Bacteroidetes* ↗  *Proteobacteria* −  Richness ↘  Shannon ↘  *Firmicutes* ↘  *Bacteroidetes* ↗  Richness ↘  Shannon ↘  Beta-diversity +  *Firmicutes* ↘  *Bacteroidetes* ↗  *Proteobacteria* –  *Verrucomicrobia*  ↗  *Firmicutes* ↘  *Bacteroidetes* ↗  *Proteobacteria* ↗  Faith’s PD ↗  Richness ↗  Shannon ↗  Beta-diversity +  Beta-diversity +  *Firmicutes* ↗  *Bacteroidetes* −  Shannon + | [1]  [2]  [3]  [1]  [4]  [5]  [6]  [7]  [8] |
| Birds  Greater sage-grouse (*Centrocercus urophasianus*)  Layer (*Gallus gallus domesticus*) | Field: Sublette (S) & Natrona (N) county, WY, USA  Commercial husbandry, Dafreng, Jiansu, China | September (S) and December (S & N), at 20°C or -3°C  May (min-max: 20.8°-25.4°C) or July (min-max: 28.6°-31.8°C) | Richness ↗  Shannon ↗  Beta-diversity +  *Firmicutes* ↘  *Bacteroidetes* ↗  *Proteobacteria* ↘  *Fusobacteria* ↘ | [9]  [10] |
| Reptiles  Common lizard (*Zootoca vivipara*)  Western fence lizard (*Sceloporus occidentalis*) | Lab  Wild-caught in lab | Present (26.6°C), intermediate (28.2°C) or warm (28.4°C) for 3 months, sampled 8 months later  Control at 25°C, experimental moved to 35°C after 7d at 25°C | *Firmicutes* ↘  *Bacteroidetes* ↘  *Proteobacteria* ↗  *Actinobacteria* ↗  Richness ↘  Beta-diversity +  *Firmicutes* ↘  Richness −  Shannon −  Beta-diversity + | [11]  [12] |
| Amphibians  Eastern red-backed salamander (*Plethodon cinereus*)  Northern leopard frog (*Lithobates pipiens*) tadpoles  Western clawed frog (*Xenopus tropicalis*) | Lab  Lab  Lab | Six days at 10°C, 15°C and 20°C  Raised at 18°C or 28°C, samples at  Gosner stage 38.3.  Raised at low (23°C) or high (28°C) temperatures, sampled as froglets, 3 days after metamorphosis | Richness ↘  Shannon ↘  Faith’s PD ↘  *Firmicutes* ↘  *Bacteroidetes* −  *Proteobacteria* ↘  *Planctomycetes* ↗  *TM6* ↗  Alpha-diversity –  Beta-diversity +  *Firmicutes*  ↘  B*acteroidetes* ↗  *Proteobacteria* ↘  *Verrucomicrobia* ↗  Richness −  Shannon −  Weighted UNIF +  Unweighted UNIF − | [13]  [14]  [15] |
| Fish  Blue tilapia (*Oreochromis aureus*)  Milkfish (*Chanos chanos*)  Rainbow trout (*Oncorhynchus mykiss*)  Yellowtail kingfish juveniles (*Seriola lalandi*) | Lab  Lab  Lab  Lab | Lines selected for cold tolerance (12°C) or not (control). Two days at 12°C or 24°C  Control (26°C) vs elevated temperature (33°C) at days 0, 14 and 21 ^b^.  11°C vs 18°C, sampled after a week  Control 24°C vs 20°C and 26°C after 30 days | *Firmicutes* −  *Bacteroidetes* ↗  *Proteobacteria* −  *Planctomycetes* ↗  *Verrucomicrobia* ↗  *TM6* ↘  Richness ↗  Shannon ↗  Beta-diversity +  *Fusobacteria* ↗  *Proteobacteria* ↘  Simpson d0 ↘  Beta-diversity +  *Firmicutes* ↘  Richness ↘  Shannon ↘  Richness ↗  Shannon ↘ | [16]  [17]  [18]  [19] |

^a^ We selected the included effects of the gut bacteria community as follows: 1) the phyla *Firmicutes* and *Bacteroidetes* were included as they are generally reported to vary with temperature or season, 2) other phyla commonly included in the analyses, were also included in the table, 3) sometimes effect directions in alpha-diversity varied between measures used, hence we report the measure type used, and 4) this differentiation did not occur in the beta-diversity, hence we did not differentiate here. Symbols: ↘, decrease with temperature; ↗, increase with temperature; −, no effect; +, significant difference. Abbreviation: PD, phylogenetic diversity. ^b^ The largest effect of a temperature increase was found at day 0, i.e., after the week required to increase the temperature from 26°C to 33°C (increased by 1°C per day).

**References to Table S3**

1. Jiang F, Gao H, Qin W, Song P, Wang H, Zhang J, et al. Marked seasonal variation in structure and function of gut microbiota in forest and alpine musk deer. Front Microbiol. 2021;12:1–13. htpps://doi.org/10.3389/fmicb.2021.699797.

2. Zhang X-Y, Sukhchuluun G, Bo T-B, Chi Q-S, Yang J-J, Chen B, et al. Huddling remodels gut microbiota to reduce energy requirements in a small mammal species during cold exposure. Microbiome. 2018;6:103. https://doi.org/10.1186/s40168-018-0473-9.

3. Bo TB, Zhang XY, Wen J, Deng K, Qin XW, Wang D-H. The microbiota–gut–brain interaction in regulating host metabolic adaptation to cold in male Brandt’s voles (*Lasiopodomys brandtii)*. ISME J. 2019;13:3037–53. https://doi.org/10.1038/s41396-019-0492-y.

4. Chevalier C, Stojanović O, Colin DJ, Suarez-Zamorano N, Tarallo V, Veyrat-Durebex C, et al. Gut microbiota orchestrates energy homeostasis during cold. Cell. 2015;163:1360–74. http://dx.doi.org/10.1016/j.cell.2015.11.004.

5. Ziȩtak M, Kovatcheva-Datchary P, Markiewicz LH, Ståhlman M, Kozak LP, Bäckhed F. Altered microbiota contributes to reduced diet-induced obesity upon cold exposure. Cell Metab. 2016;23:1216–23. https://doi.org /10.1016/j.cmet.2016.05.001.

6. Worthmann A, John C, Rühlemann MC, Baguhl M, Heinsen FA, Schaltenberg N, et al. Cold-induced conversion of cholesterol to bile acids in mice shapes the gut microbiome and promotes adaptive thermogenesis. Nat Med. 2017;23:839–49. https://doi.org/10.1038/nm.4357.

7. Liu P-Y, Cheng A-C, Huang S-W, Chang H-W, Oshida T, Yu H-T. Variations in gut microbiota of Siberian flying squirrels correspond to seasonal phenological changes in their Hokkaido subarctic forest ecosystem. Microb Ecol. 2019;78:223–31. https://doi.org/10.1007/s00248-018-1278-x.

8. Zhu Z, Sun Y, Zhu F, Liu Z, Pan R, Teng L, Guo S. Seasonal variation and sexual dimorphism of the microbiota in wild blue sheep (*Pseudois nayaur*). Front Microbiol. 2020;11:1260. https:/doi.org/10.3389/fmicb.2020.01260.

9. Drovetski SV, O’Mahoney MJV, Matterson KO, Schmidt BK, Graves GR. Distinct microbiotas of anatomical gut regions display idiosyncratic seasonal variation in an avian folivore. Anim Microbiome. 2019;1:1–11. https://doi.org/10.1186/s42523-019-0002-6.

10. Zhu L, Liao R, Wu N, Zhu G, Yang C. Heat stress mediates changes in fecal microbiome and functional pathways of laying hens. Appl Microbiol Biotechnol. 2019;103:461–72. https://doi.org/10.1007/s00253-018-9465-8.

11. Bestion E, Jacob S, Zinger L, Di Gesu L, Richard M, White J, et al. Climate warming reduces gut microbiota diversity in a vertebrate ectotherm. Nat Ecol Evol. 2017;1:1–3. https://doi.org/ 10.1038/s41559-017-0161.

12. Moeller AH, Ivey K, Cornwall MB, Herr K, Rede J, Taylor EN, et al. Lizard gut microbiome changes with temperature and is associated with heat tolerance. Appl Environ Microbiol. 2020; 86:e01181-20. https://org.doi/10.1128/AEM.01181-20.

13. Fontaine SS, Novarro AJ, Kohl KD. Environmental temperature alters the digestive performance and gut microbiota of a terrestrial amphibian. J Exp Biol. 2018;221:jeb187559. https://doi.org/10.1242/jeb.187559.

14. Kohl KD, Yahn J. Effects of environmental temperature on the gut microbial communities of tadpoles. Environ Microbiol. 2016;18:1561–5. https://doi.org/10.1111/1462-2920.13255.

15. Li J, Rui J, Li Y, Tang N, Zhan S, Jiang J, et al. Ambient temperature alters body size and gut microbiota of *Xenopus tropicalis*. Sci China Life Sci. 2020;63:915–25. https://doi.org/10.1007/s114217-019-9540-y.

16. Kokou F, Sasson G, Nitzan T, Doron-Faigenboim A, Harpaz S, Cnaani A, et al. Host genetic selection for cold tolerance shapes microbiome composition and modulates its response to temperature. eLife. 2018;7:e36398.

https://doi.org/10.7554/eLife.36398.001.

17. Hassenrück C, Reinwald H, Kunzmann A, Tiedemann I, Gärdes A. Effects of thermal stress on the gut microbiome of juvenile milkfish (*Chanos chanos*). Microorganisms. 2021;9:5. ttps://dx.doi.org/10.3390/microorganisms9010005.

18. Huyben D, Sun L, Moccia R, Kiessling A, Dicksved J, Lundh T. Dietary live yeast and increased water temperature influence the gut microbiota of rainbow trout. J Appl Microbiol. 2018;124:1377–92. https://doi.org/10.1111/jam.13738.

19. Soriano EL, Ramírez DT, Araujo DR, Gómez-Gil B, Castro LI, Sánchez CG. Effect of temperature and dietary lipid proportion on gut microbiota in yellowtail kingfish *Seriola lalandi* juveniles. Aquaculture. 2018;497:269–77. https://doi.org/10.1016/j.aquaculture.2018.07.065.


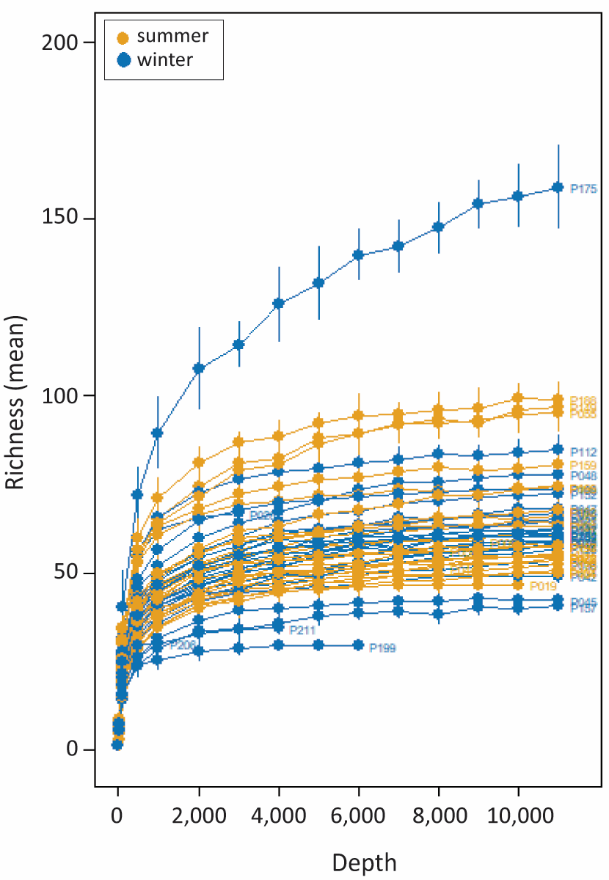


**Fig. S1** Richness rarefaction curves. Most curves levelled off around 3,000 reads.


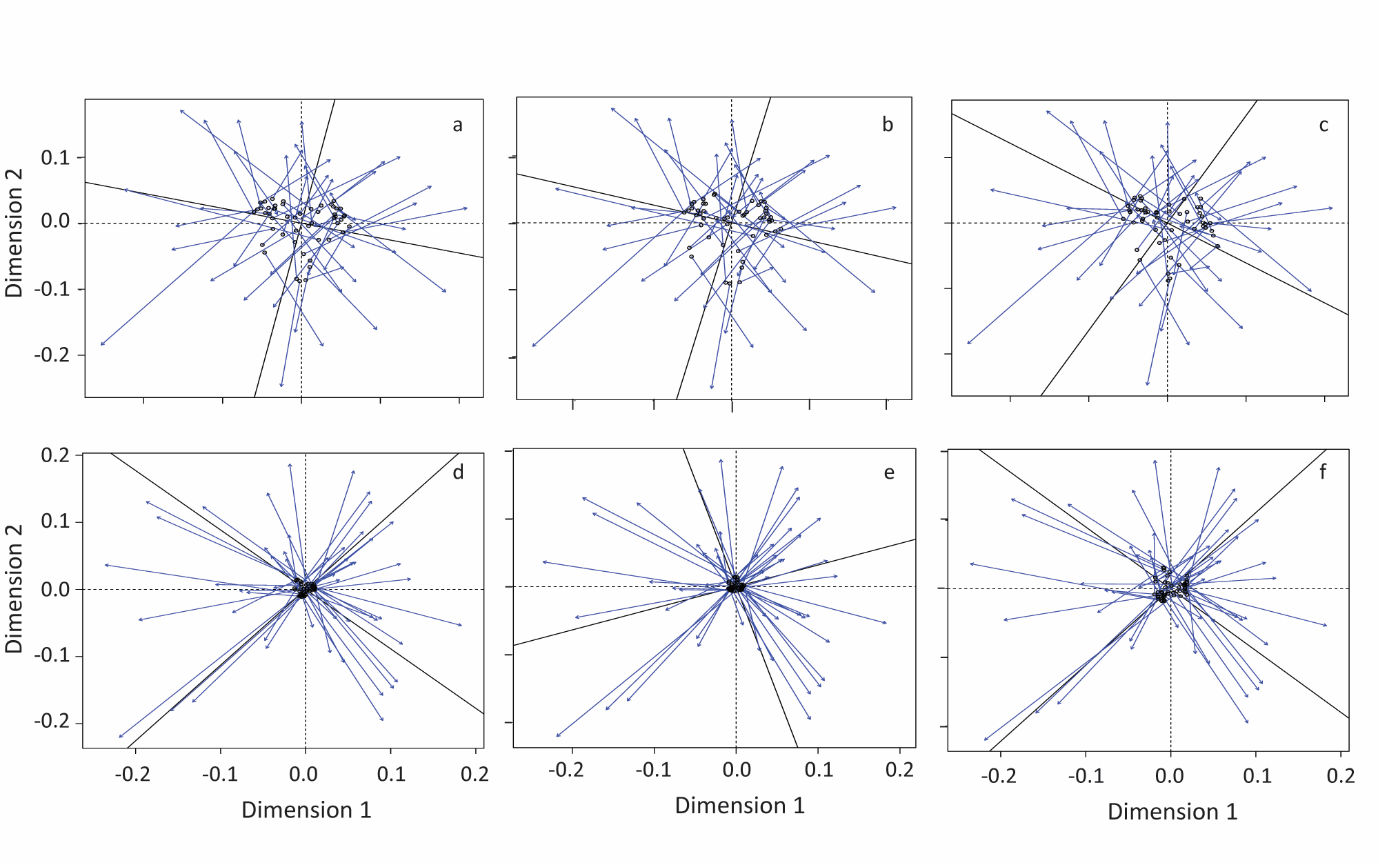


**Fig. S2** Results of the Procrustes analyses. The ordination dimensions of the metabolism indices versus the ordination dimensions of the Jaccard (**a**), Brays-Curtis (**b**) and weighted UniFrac (dis)similarities and distances (**c**). And the ordination dimensions of the seven innate immune indices versus the ordination dimensions of the Jaccard (**d**), Brays-Curtis (**e**) and weighted UniFrac (**f**).


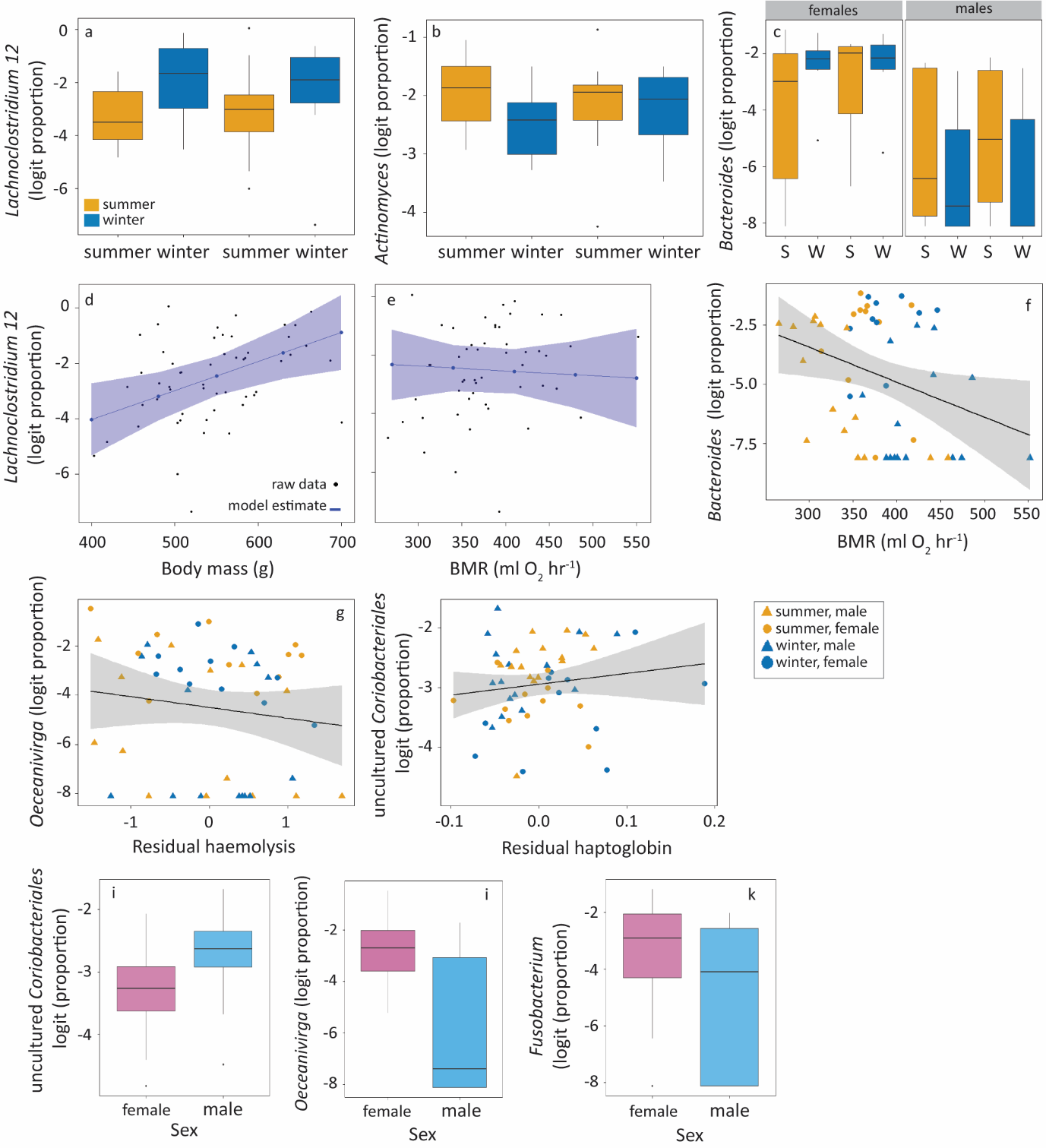


**Fig. S3** Significant variation in the Logit-transformed proportions of the most abundant genera with season, sex, metabolism or immune indices. Boxplots of the seasonal variation in *Lachnoclostridum12* (**a**), *Actinomyces* (**b**), and *Bacteroides* (**c**), for the latter per sex. Seasons (S = summer, W = winter) start with the summer of 2013. The model estimates for the linear relationships between *Lachnoclostridum12* and BMR and body mass are presented in panels (**d**, **e**). Logit-transformed proportions of *Bacteroides* increased with increasing BMR (**f**). *Oceanivirga* decreased with residual haemolysis (**g**) and uncultured *Coriobacterium* increased with increasing residual haptoglobin (**h**). The uncultured *Coriobacterium*, *Oceanivirga* and *Fusobacterium* also varied with sex (**i**, **j**, **k**). Statistics are presented in Table 3 of the main text.

**
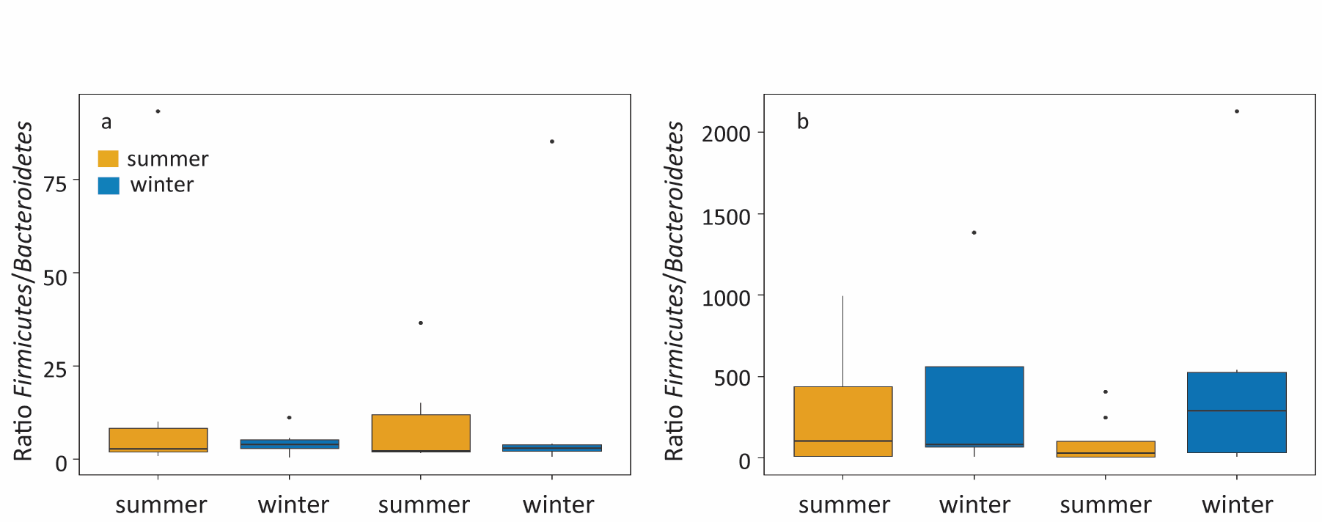
**

**Fig. S4** Seasonal variation in the *Firmicutes*:*Bacteroidetes* ratio in (**a**) females and (**b**) males. There was a significant effect of the interaction season*sex on the *Firmicutes*:*Bacteroidetes* ratio (LMM, *F*_1,31_ = 4.79, *P* = 0.04, aviary did contribute significantly to the model, *P* = 0.01). Note the different ranges of the y-axes.
